# Indenting at the Microscale: Guidelines for Robust Mechanical Characterization of Alginate Microgels

**DOI:** 10.1021/acsami.4c20952

**Published:** 2025-02-24

**Authors:** Philipp Harder, Leonard Funke, Jana Tamara Reh, Oliver Lieleg, Berna Özkale

**Affiliations:** † Microrobotic Bioengineering Lab (MRBL), School of Computation Information and Technology, Technical University of Munich, Hans-Piloty-Straße 1, 85748 Garching, Germany; ‡ Munich Institute of Robotics and Machine Intelligence, Technical University of Munich, Georg-Brauchle-Ring 60, 80992 Munich, Germany; § Munich Institute of Biomedical Engineering, Technical University of Munich, Boltzmannstraße 11, 85748 Garching, Germany; ∥ TUM School of Engineering and Design, Department of Materials Engineering, Technical University of Munich, Boltzmannstraße 15, 85748 Garching, Germany; ⊥ Center for Protein Assemblies (CPA), Technical University of Munich, Ernst-Otto-Fischer-Str. 8, 85748 Garching, Germany

**Keywords:** microgels, alginate, ionic cross-linking, nanoindentation, Young’s modulus

## Abstract

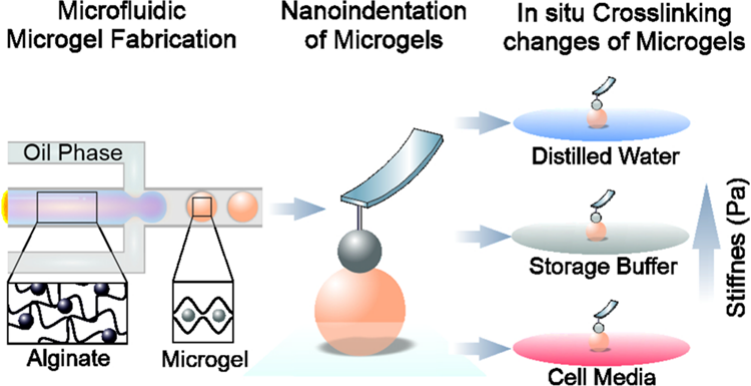

Microgels offer broad applications in bioengineering due to their customizable properties, supporting innovations in mechanobiology, tissue engineering, drug delivery, and cell therapy. This study focuses on characterizing ionically cross-linked alginate microgels using a nanoindentation technique, enabling precise assessment of their mechanical properties at the microscale. We report on the microfluidic fabrication of alginate microgels with varying sizes at different cross-linker concentrations and on the mechanical characterization of the resulting microgels in terms of Young’s moduli as well as viscoelastic behavior. Measurements conducted using dynamic nanoindentation reveal that microgel elasticity is strongly influenced by the ionic composition of the surrounding media, in particular, the concentration of calcium and sodium. We demonstrate that the highest Young’s modulus observed for ionically cross-linked alginate microgels is in deionized water (7.2 ± 0.9 kPa). A drastic softening effect is observed when the calcium cross-linked microgels are placed into a storage buffer containing divalent ions (0.7 ± 0.1 kPa) and cell culture media consisting of Dulbecco’s Modified Eagle Medium (0.2 ± 0.1 kPa) with fetal bovine serum (0.4 ± 0.1 kPa). High concentrations of sodium were found to disrupt ionic cross-links, decreasing stiffness and increasing viscosity, with reversible effects observed upon switching back to deionized water. These findings highlight the importance of media selection for applications requiring mechanical stability, and we provide guidelines for measuring the mechanical properties of microgels in a robust manner that is applicable to a wide range of different conditions.

## Introduction

Microgels have emerged as a group of versatile tools in bioengineering, enabling a variety of applications in mechanobiology, tissue engineering *in vitro*, on-demand drug delivery, and therapeutic cell transplantation *in vivo.*(1−9) Both natural and synthetic hydrogels have been used to generate microgels with tailored properties. For example, polyethylene glycol (PEG) based microgels, decorated with cell-adhesion ligands, have been used as building blocks to generate three-dimensional (3D) cell-carrying scaffolds with precisely regulated porosity and intracellular spacing.^10,11^ The modularity of this approach has enabled spatial patterning in the assembled scaffolds thus allowing investigations on immune cell behavior and stem cell differentiation in 3D.^2,11^ In parallel, alginate- and polyacrylamide-based microgels have been used as stress sensors in tumor spheroids, enabling a real-time measurement of cell-generated traction forces at a microscale resolution in 3D cell culture environments.^12,13^ Microfluidic strategies have been used to directly encapsulate individual mammalian cells into alginate microgels, which acted as therapeutic cargo carriers *in vivo.*(14) Transplanting alginate-encapsulated stem cells in a mouse model increased their circulation time in vivo by at least 2 orders of magnitude, leading to a drastic increase in the therapeutic performance of their immunomodulatory properties.^15,16^ These examples clearly demonstrate the versatility of the microgels.

Among the various microgel systems, alginate-based microgels have been widely used due to their tunable chemical and mechanical properties, excellent biocompatibility, and ease of microfluidic production.^17,18^ Implementing established carbodiimide chemistry on the biopolymer has allowed the conjugation of cell adhesion ligands, fluorescent tracking molecules, and small cell-secreted proteins.^19−21^ Owing to its chemical adaptability, alginate has been used to culture a variety of mammalian cells including but not limited to macrophages, fibroblasts, and stem cells in 3D constructs.^11,22−26^ Changing the cross-linking mechanism and molecular weight of alginate has similarly enabled control over its mechanical properties such as porosity, elastic modulus, and stress relaxation at the macroscale.^27,28^ For example, ionically cross-linking alginate with increasing concentrations of divalent ions (e.g., Ca^2+^) allowed control over Young’s moduli within a range of 1–550 kPa.^25,29^ Integrating other polymers such as polyethylene glycol into ionically cross-linked alginate enabled precise regulation over stress relaxation processes, independent of the elastic modulus.^22^ Ionic cross-linking has been particularly advantageous due to its compatibility with the microfluidic production of microgels. The process commonly involves the generation of droplets carrying uncross-linked alginate and nanoparticles, which release divalent ions (e.g., Ca^2+^, Ba^2+^, Sr^2+^) to cross-link alginate chains when the aqueous stream comes into contact with the oil phase carrying a dilute acid.^30^ A variety of alginate microgels with application-specific properties have been engineered with this approach. Despite these advances, the range and regulation of mechanical tunability remain limited at the microscale.

Part of the challenge here is effectively measuring the mechanical properties of microgels in a reliable manner. Measurements at the macroscale have traditionally relied on rheological methods to characterize the elastic moduli and the viscosity of alginate hydrogels.^31,32^ The influence of molecular weight, choice of cross-linking method, and the degree of cross-linking on the viscoelastic properties of macroscale alginate hydrogels have been investigated in detail.^31,33,34^ As a result, reliable protocols on how to conduct mechanical characterization and prepare precise formulations to reach specific viscoelastic properties in alginate gels have been established.^27,35^ The application of surface-based techniques such as atomic force microscopy (AFM) and nanoindentation further corroborated these findings, while providing new methods to investigate local changes in the surface properties of macroscale alginate hydrogels.^36,37^ In contrast, methods established for macroscale systems are often incompatible with microgels due to the small size of the structures in question. Strategies relying on AFM have been implemented instead to mechanically characterize microgels. However, interpreting the measurements is often challenging due to undesirable probe-sample interactions, leading to surface remodeling and chemical instability of microgels in changing aqueous environments.^38−40^ The latter is particularly relevant for ionically cross-linked alginate microgels, due to the dynamic cross-links that are susceptible to the presence of other ionic species. There is currently a lack of reliable measurement strategies compatible with microgels, and addressing this challenge is necessary to further advance their capabilities.

Recognizing this need, we developed a cantilever-based mechanical characterization method using ionically cross-linked alginate microgels. A commercially available stage-top nanoindentation system, the Chiaro Nanoindenter from Optics 11, coupled with a fluorescent microscope, was employed to conduct indentation on the microgels. The system allowed *in situ* visualization and tracking of the microgels throughout the measurements. Utilizing microfluidics, we fabricated alginate microgels with exceptional uniformity that served as a reproducible and reliable benchmark for mechanical testing. This approach enabled the production of homogeneous microgels, and different species of microgels with average sizes ranging from 26.0 ± 2.9 to 36.3 ± 1.3 μm were achieved by changing the microfluidic channel geometry. We then systematically applied *in situ* nanoindentation measurements to determine the Young’s moduli of alginate microgels and conducted dynamic mechanical analysis (DMA) to map their viscoelastic properties. Overall, our measurements revealed a strong influence of cross-linker concentration and ionic strength of the aqueous media on the elastic moduli of alginate microgels, which varied from 0.2 ± 0.1 to 19.9 ± 3.0 kPa. A wide range of parameters was tested to understand the potential influence of sample-to-substrate attachment, probe size and stiffness, and indentation depth and speed on the measured elastic moduli. While sample preparation and indentation parameters significantly influenced the measurements, the probe size did not affect the results. Based on our results, we provide a list of guidelines for reliable characterization of microgels.

## Methods and Materials

### Preparation of Microfluidic Devices

Microfluidic devices with channel geometries ranging from 10 to 30 μm were fabricated using soft lithography with SU-8 3050 photoresist (Kayaku Advanced Materials), as previously described.^14,41^ The spin coating process involved two steps: first, the wafer was spun at 500 rpm for 10 s with an acceleration of 100 rpm/s, followed by a second spin coating step at 4000 rpm for 30 s with an acceleration of 300 rpm/s to achieve a uniform 25 μm thick layer. It was then soft-baked at 95 °C for 15 min. The UV exposure dose was maintained at 250 μJ/cm^2^, and the samples were baked at 68 °C for 2 min, followed by a gradual ramping of the temperature over 15 min to 95 °C. The temperature was then maintained at 95 °C for 5 min to completely cure the photoresist. Once the master was developed, polydimethylsiloxane (PDMS, Dow Corning) was mixed with the cross-linker at a 10:1 ratio. The PDMS mixture was degassed, poured onto the master, and cured at 65 °C for at least 1 h. After curing, the PDMS layer was carefully removed from the master, and inlets and outlets were punched using a 1 mm biopsy punch (Kai Medical). The negative imprint was bonded to a glass surface using plasma ashing (Piezobrush PZ3, Relyon Plasma GmbH). The devices were flushed with a water-repellent chemical (Rain-X) to enhance the fluid flow.

### Alginate Preparation

High molecular weight alginate (I-1G, 280 kDa) was obtained from KIMICA and used as the base material for preparing three types of functionalized alginate: RGD-functionalized alginate, Rhodamine B-functionalized alginate, and biotin-functionalized alginate. For the functionalization processes, carbodiimide chemistry was employed using 1-ethyl-3-(3-(dimethylamino)propyl) carbodiimide (EDC) and *N*-hydroxysulfosuccinimide (sulfo-NHS) (Thermo Fisher Scientific). The alginate was dissolved in 2-(N-morpholino)ethanesulfonic acid (MES) buffer at a 1% concentration. For RGD functionalization, the integrin-binding RGD peptide (Gly)4–Arg–Gly–Asp–Ser–Pro (GGGGRGDSP, Peptide 2.0) was added to the solution at a degree of substitution (DS) of 20 relative to the molar amount of alginate, based on a previously established protocol.^8^ The amount of RGD in RGD-modified alginate was targeted to be 71.4 mM, calculated based on the desired substitution level and the molecular weight of alginate (280 000 g/mol). To achieve this, 142.31 mg of RGD peptide (63.5% purity, molecular weight 758.75 g/mol) was added per gram of alginate. Conjugation was facilitated assuming a coupling efficiency of 60%, using solutions of sulfo-NHS and EDC in MES buffer, which were prepared fresh at final concentrations of 1.26 mM (274 mg/1 g alginate) and 2.53 mM (484.2 mg/1 g alginate), respectively. For fluorescent labeling, functionalization was performed using Rhodamine B Lissamine (Thermo Fisher Scientific) to target a DS of 2, following the same procedure. For the biotin functionalization, EZ-Link Amine-PEG3-Biotin (Thermo Fisher Scientific) was added to the alginate solution at a DS of 20 similarly. The reaction was allowed to proceed for 20 h at room temperature in the presence of EDC and sulfo-NHS, facilitating the covalent attachment of the functional groups to the alginate backbone. All three types of alginates were prepared separately. To purify these alginates, we dialyzed them against decreasing concentrations of sodium chloride for 3 days, followed by treatment with activated charcoal. The purified alginate solution was then filtered through a 0.22 μm membrane and lyophilized at −50 °C for 1 week using a benchtop freeze-dryer (FreeZone 4.5L, Labconco, USA). The lyophilized alginate was stored at −20 °C until further use.

### Preparation of Calcium Carbonate (CaCO_3_) Nanoparticles

Prior to use, CaCO_3_ nanoparticles (CalEssence, PCC70) were sterilized using an autoclave at 121 °C for 20 min (Varioklav). Sterile CaCO_3_ (PCC70) was weighed to 14–16 mg and placed in a 1.7 mL microcentrifuge tube. The CaCO_3_ was suspended in 600 μL of Dulbecco’s Modified Eagle Medium (DMEM) and sonicated for 15 s at 70% amplitude (Fisherbrand Model 120 Sonic Dismembrator). The probe was wiped with ethanol before use to ensure proper sonication. The CaCO_3_ suspension was diluted with 23 mL of DMEM and centrifuged at 50 rcf for 5 min at 20 °C. The top 20 mL of the supernatant was collected and subjected to a second centrifugation at 1000 rcf for 5 min. The supernatant containing dissolved particles was aspirated using a 200 μL pipet tip attached to an aspirator. The remaining particles were resuspended in complete DMEM to achieve a 10 mg/mL (99,91 mM) concentration, resulting in a 1.5 mL final volume.

### Microgel Fabrication

Two types of microgels were fabricated in this study: RhB-functionalized alginate carrying microgels (M1) and plasmonic gold nanorod (Au-nanorod) loaded microgels (M2). To begin, dried alginate was reconstituted at 2 wt % in MES buffer. RhB microgels were prepared by using RhB-functionalized alginate. For AuNP microgels, a mixture of 80% RGD-functionalized alginate and 20% biotin-functionalized alginate was used, with gold nanorods added to a final concentration of 4.875 mg/mL. This mixture was tip-sonicated for 15 s at 60% amplitude using a Fisherbrand Model 120 Sonic Dismembrator to ensure homogeneous dispersion of the nanorods. The microfluidic setup consisted of a microfluidic chip with a cross-junction design^41^ and syringes holding the aqueous and oil phases, connected via polytetrafluoroethylene (PTFE, VWR) tubing. The aqueous phase consisted of the previously prepared alginate mixtures (1 wt %), CaCO_3_ nanoparticles (20.8 mM), and a buffer solution medium comprising 130 mM NaCl, 2 mM CaCl_2_, and 25 mM HEPES, referred to as bead buffer (BB). The oil phase consisted of fluorinated oil HFE 7500 (Novec 7500 engineered fluid, 3M), fluorinated surfactant Pico-Surf (Sphere fluidics), and 0.037 vol % acetic acid (Sigma). The acetic acid in the oil phase dissolves the CaCO_3_ nanoparticles following the pinching-off process at the junction site, releasing calcium (Ca^2+^) ions to cross-link the alginate. The cross-linker concentrations of 21.8 and 40.5 mM Ca^2+^ were calculated based on the initial amount of CaCO_3_ added during the cross-linking process in the presence of 2 mM CaCl_2_-containing bead buffer. All CaCO_3_ nanoparticles present in the droplets were assumed to completely dissolve. The total Ca^2+^ concentration per microfluidic fabrication process was calculated by summing the Ca^2+^ contribution from the bead buffer and the Ca^2+^ released from dissolved CaCO_3_, considering the experimental volumes. The resulting concentrations were calculated as 3.1 mM for prepolymer solutions containing 5 μL of CaCO_3_ solution, 21.8 mM for 50 μL CaCO_3_ solution, and 40.5 mM for 95 μL CaCO_3_ solution. The aqueous phase was loaded into a 1 mL syringe, while the oil phase was placed into a 3 mL syringe. Both syringes were loaded onto a single syringe pump (Darwin Microfluidics), and the microfluidic process was conducted at flow rates of 2, 4, 6, 8, and 10 μL/min. The resulting microgels were collected and demulsified using 1H,1H,2H,2H-perfluoro-1-octanol (PFO, Sigma), and stored in water or bead buffer at 4 °C for further use.

### Microscope Setup and Imaging Analysis

Experiments were conducted using a Leica DMI8 inverted microscope with 5, 10, and 40× air objectives. A 16-bit black and white CCD camera (Leica) was used for image acquisition. Leica LAX software was used to operate the microscope and conduct fluorescent imaging. The exposure time for the microgel size analysis was set to 300 ms for fluorescent microgels and 50 ms for bright-field imaging across all samples. The open-source Fiji ImageJ2 software was used to quantify the size of all microgels, where an automatic thresholding method was used to analyze the fluorescent images of RhB-microgels, and circumference detection was done manually on microgels without fluorescent alginate by relying on brightfield images.

### Rheological Measurements and Macrogel Fabrication

All rheological measurements were performed using a commercial shear rheometer (MCR302, Anton Paar) equipped with a planar bottom plate (P-PTD200/56, Anton Paar) and a parallel-plate measuring geometry (PP25, Anton Paar). For time-dependent measurements, the storage modulus (*G*′) and loss modulus (*G*″) were recorded at a fixed frequency of 1 Hz over a duration of 10 min, with a data acquisition interval of 30 s. The plate separation was set to 400 μm with a sample volume of 240 μL, and measurements were conducted at room temperature. To prepare M1-type macroscale prepolymer mixtures, 2 wt % of RhB alginate was diluted to 1 wt % with CaCO_3_ nanoparticles and bead buffer (final concentration 21.8 mM Ca^2+^ or 40.5 mM Ca^2+^). Immediately after mixing, the viscoelastic response of the material was assessed by recording the viscoelastic moduli over time in torque-controlled mode (*M* = 0.5 μN·m). Additionally, frequency-dependent measurements were conducted over a range of 0.1 and 10 Hz to determine the dynamic mechanical properties of macroscale alginate gels. Before every frequency sweep, a pre-experiment was carried out to guarantee a linear material response, after which the shear strain was set to 1.5 times the average shear strain obtained in 5 repetitions at an oscillatory torque of 0.5 μN·m. M1 alginate macrogels were then fabricated, containing 500 μL of 2 wt % RhB alginate, diluted to 1 wt % by adding CaCO_3_ nanoparticles and bead buffer. Two cross-linking strategies were followed, one involved the passive dissolution of CaCO_3_ nanoparticles via 0.037% acetic acid in surrounding media, and the other relied on the actively initiated dissolution of the cross-linker particles using 200 mg/mL glucono-δ-lactone (GDL, Sigma-Aldrich) in the prepolymer mixture. To prepare passively cross-linked M1-type macrogels, 2 wt % of RhB alginate was diluted to 1 wt %, with CaCO_3_ nanoparticles and bead buffer (final concentration 21.8 mM Ca^2+^), and left to cross-link in HFE 7500 oil (Novec 7500 engineered fluid, 3M) containing 0.037 vol % acetic acid (Sigma) over 12 h. For GDL-cross-linked M1 macrogels, 200 mg/mL GDL was added to 2 wt % RhB alginate in addition to CaCO_3_ and bead buffer (final concentration 21.8 or 40.5 mM Ca^2+^). After mixing for 30 s, GDL gradually released calcium ions, resulting in gel formation over 12 h.

### Nanoindentation

The mechanical properties of all microgels were investigated using a microscope-compatible stage-top nanoindenter (Chiaro, Optics11 Life). Probes with tip sizes of 3 and 11 μm, and stiffness values of 0.5 and 0.025 N/m, were used for all nanoindentation measurements. Specifically, combinations of 3 μm and 0.54 N/m, 11 μm and 0.55 N/m, 3 μm and 0.021 N/m, and 11 μm and 0.018 N/m were utilized. Indentation was performed to a depth of 1000 nm at an indentation speed of 1250 nm/s. Dynamic mechanical analysis (DMA) was performed with an indentation depth of 10000 nm for viscoelastic measurements to ensure a measurable response. At this depth, the indentation tip oscillated at the reported frequencies with an amplitude of 100 nm for 5 periods. Prior to nanoindentation measurements, microgels were adhered to the bottom of the well plate by using poly-l-lysine (PLL, Merck) to prevent movement during testing. For this purpose, PLL was dissolved in water at concentrations of 0.05, 0.1, and 0.5 mg/mL. Then, 3 μL of dissolved PLL was added to the well plate surface. The PLL-treated well plate was then placed on a hot plate at 35 °C for 30 min. Any excess PLL was removed by gently pipetting off the remaining liquid to ensure complete drying. After drying, microgels were seeded into the PLL-treated area with the addition of deionized water, microgel storage media, or cell media. After sufficient time was allowed for the microgels to settle and adhere to the PLL-coated surface, nanoindentation measurements were performed. The force–indentation curves were fitted using the Hertzian contact model via the Optics11 Data Viewer software. The effective elastic modulus (*E*_eff_) was calculated using eq 1,
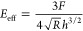
1where *F* is the applied load, *R* is the indenter tip radius, and *h* is the maximum indentation depth. Assuming a Poisson’s ratio of ν = 0.49, Young’s modulus was finally calculated using eq 2,
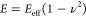
2The Hertzian model was applied to the loading curve, utilizing the Optics 11 analysis software. 90% of the maximum force detected during the indentation was used to ensure a reliable fit. The Hertzian model was selected based on its suitability for soft tissues within the 0–35 kPa range.^42−44^ Additionally, Dynamic Mechanical Analysis (DMA) was employed to characterize the viscoelastic properties of alginate-based microgels, specifically assessing their suitability for applications in drug delivery and stimuli-responsive systems.^45^ Key parameters measured included the storage modulus (*E*′), indicative of elastic response, and the loss modulus (*E*″), which reflects energy dissipation. The phase angle (δ) also provided insight into the balance between elastic and viscous responses.^46^ The experimental setup involved nanoindentation with an M2-type microgel configuration using an indentation tip lowered to a 10 μm depth to ensure adequate engagement with the microgel surface. Oscillatory testing was conducted at a peak amplitude of 100 nm across frequencies of 1, 2, 4, and 10 Hz, with a relaxation interval of 2 s between frequency steps.

### Statistical Analysis

Data are presented as means ± standard deviation, based on at least three independent measurements. Statistical testing for paired data utilized a paired Student’s *t-*test following confirmation of normal distribution. When the data was not normally distributed, the Wilcoxon Signed-Rank Test was applied for paired comparisons. For comparisons involving multiple groups, one-way ANOVA was employed, with Tukey’s test used for posthoc analysis to identify specific group differences. Two-way ANOVA was performed to evaluate both main effects and interactions when assessing the effects of multiple factors. Statistical significance thresholds were set to nonsignificant (ns), *P < 0.05, **P < 0.01, and ***P < 0.001. Analyses and graphing were carried out using OriginPro2021b.

## Results and Discussion

### Fabrication of Alginate Microgels with Varying Size and Cross-linking Density

We used a previously established microfluidic platform to fabricate alginate microgels^18^ and produced different groups of alginate-based microgels with varying sizes and compositions. We chose this approach because of its proven ability to control key fabrication parameters precisely.^41,47^ Our primary goal was to understand how parameters such as microgel size, cross-linker concentration, the presence of different functional moieties, the degree of available cross-linking sites on alginate chains, and sample preparation affect the mechanical properties of alginate microgels. Considering the wide range of applications of such microgels, it was crucial to investigate the influence of different formulations on the nanoindentation measurements.

**Figure 1:**
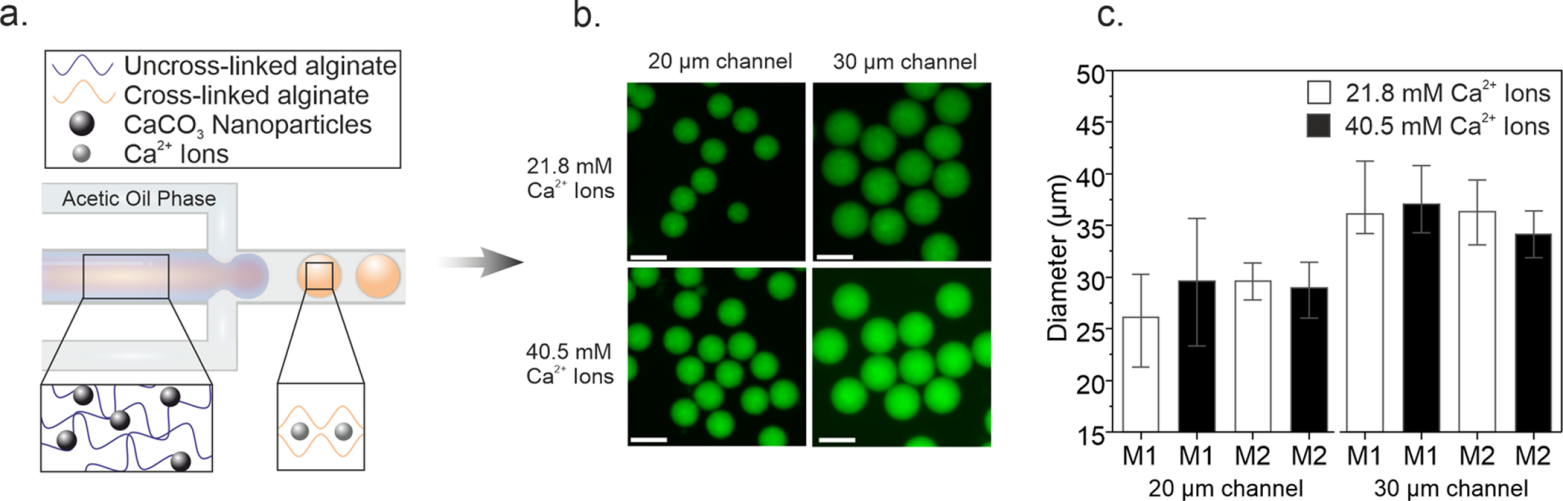
Microfluidic fabrication of alginate microgels. (a) Schematic diagram of microfluidic fabrication of homogeneous alginate microgels, using acetic acid containing oil phase. The prepolymer mixture consisting of uncross-linked alginate and CaCO_3_ nanoparticles is pinched off at the T-junction site. The subsequent dissolution of CaCO_3_ nanoparticles enables free Ca^2+^ cations that cross-link the microgel droplets. (b) Representative images of M1-type microgels consisting of RhB-functionalized alginate, sorted according to Ca^2+^ cross-linker concentration and channel size (scale bars: 30 μm). (c) Analysis of microgel size for M1- and M2- type microgels is plotted for two different cross-linker concentrations in 20 and 30 μm channel devices (*n* = 400 for M1, *n* = 100 for M2).

The microfluidic fabrication process involved the flow of two immiscible streams, where the aqueous phase carried alginate solution and the cross-linker CaCO_3_ nanoparticles, while the oil phase carried a weak acid (Figure 1a). Ionic cross-linking was briefly initiated when mixing alginate solutions with CaCO_3_ nanoparticles due to the spontaneous dissolution of the particles (Figure S1). However, once droplets were formed at the T-junction site, the acetic acid in the oil phase completely dissolved the CaCO_3_ nanoparticles, enabling the release of all free Ca^2+^, which simultaneously finalized the cross-linking of alginate chains. Using this approach and by varying the microfluidic channel dimensions, we produced microgels consisting of RhB-functionalized alginate (M1) with different sizes (Figure 1b). Including the fluorescent molecule RhB in the alginate microgels enabled the detection and tracking of microgels during imaging that were otherwise completely transparent and indiscernible in brightfield mode. We chose a relatively low DS value to keep the alginate network as close as possible to its unmodified state. The resulting M1-type alginate microgels exhibited a high signal-to-noise ratio during fluorescence microscopy, making it easy to visualize and track them during imaging (Figure 1b). M1 microgels fabricated using a 20 μm-wide channel at 21.8 mM of Ca^2+^ concentration had an average diameter of 26.0 ± 2.9 μm, whereas increasing the channel size to 30 μm with the same recipe resulted in microgels with an average diameter of 36.1 ± 4.1 μm. Increasing the concentration of Ca^2+^ to 40.5 mM, while maintaining the channel size at 20 μm, led to a slight increase in microgel diameter of 29.0 ± 2.5 μm. Utilizing the same recipe and microfluidic devices with a 30 μm channel size yielded M1 microgels with a diameter of 37.0 ± 1.3 μm. All M1-type microgels exhibited uniform fluorescence, although increasing the concentration of free Ca^2+^ led to microgels with brighter RhB signal, most likely due to improved inclusion of the RhB-alginate during cross-linking. The reported calcium ion concentrations are based on the theoretical assumption of complete uptake and cross-linking. However, in practice, cross-linking efficiency may be affected by factors such as ion diffusion limitations, steric hindrance, and reaction kinetics. When the concentration of Ca^2+^ ions in the aqueous phase was reduced to 3.1 mM within the 30 μm channel, the resulting microgels exhibited non-uniform morphology with poorly defined edges (Figure S2). This finding indicated that the cross-linker concentration necessary to maintain microgel integrity was above 3.1 mM. Systematically increasing the flow rate during fabrication from 2 to 10 μL/min in a 30 μm channel device resulted in smaller microgels (31.4 ± 2.9 μm) at 8 μL/min (Figure S3). In contrast, microgels fabricated at all other flow rates revealed similar average sizes (Figure S3). This finding may have resulted from using a single syringe pump for processing solutions at different speeds due to the difference in the syringe sizes used for the aqueous and oil phases. At a flow rate of 8 μL/min, minor discrepancies in the flow rates were likely introduced by each syringe despite a constant speed setting, potentially leading to increased localized shear forces or hydrodynamic effects, resulting in smaller microgel sizes. Conversely, this mismatch may have been mitigated at higher flow rates.

We next sought to understand the influence of differences in microgel formulation on the morphology of the resulting microgels. Integrating biomolecules, peptides, and stimuli-responsive nanoparticles has enabled a wide variety of applications such as stem cell culture, tissue engineering, and microrobotics.^41,47,48^ However, the influence of changes in alginate modification and the inclusion of metallic nanoparticles on the mechanical properties of the resulting microgels is unknown. To address this, we fabricated M2-type microgels consisting of heavily functionalized alginates with biotin, the cell-binding RGD peptides, and plasmonic gold (Au) nanoparticles (Figure S4). The presence of Au-nanoparticles within the microgels had a minimal impact on the size distribution (Figure 1c). M2-type microgels, produced using 20 μm channels and a Ca^2+^ concentration of 21.8 mM, had an average diameter of 29.6 ± 0.9 μm, compared to 26.0 ± 2.9 μm for nonfunctionalized microgels—a difference of approximately 13.8% (Figure 1c). Similarly, M2-type microgels fabricated using a 30 μm channel width, under the same cross-linker conditions, were found to have an average diameter of 36.3 ± 1.3 μm, a size difference of 0.55% compared to M1-type microgels. Taken together, these findings showed that the primary factor in determining microgel size was the channel size, while differences in formulation and cross-linker concentration did not necessarily influence microgel size.

### Impact of Size and Ionic Cross-Linking on Microgel Stiffness

**Figure 2:**
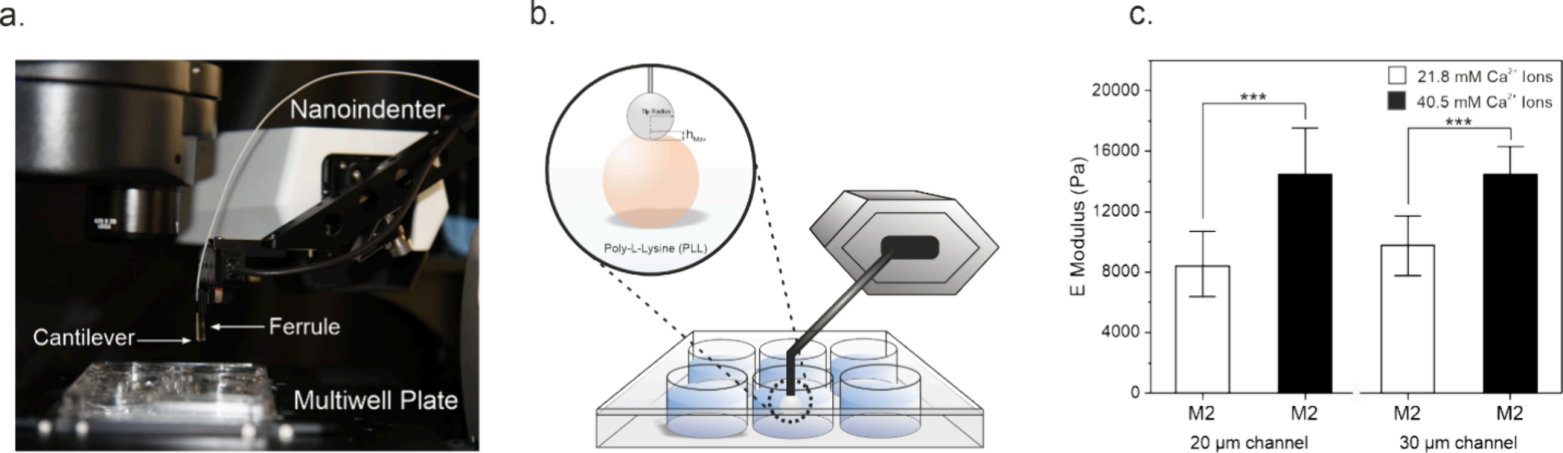
Mechanical characterization of alginate microgels using nanoindentation. (a) Real-life image of the nanoindentation system is shown, indicating the nanoindenter arm, ferrule, and the attached cantilever. Microgels are placed in a multiwell plate for the measurements. (b) Schematic of the nanoindentation setup using a 6-well plate, with Poly-l-Lysine (PLL) as a substrate coating, nanoindentation probe depicted in gray, microgel depicted in orange with *h*_max_ indicating maximum indentation depth. (c) Young’s moduli of M2-type microgels, with varying sizes and cross-linker amounts (*n* = 30 per condition, ***P < 0.001, two-way ANOVA).

Having characterized the size and morphology of the microgels, we focused our efforts on investigating the mechanical properties of the microgels using a probe-based approach. We used a stage-top nanoindenter coupled to a fluorescence microscope to conduct the indentation measurements on microgels placed in a six-well plate (Figure 2a).

Prior to measurements, the surface of the well plates was coated with a thin layer of Poly-l-Lysine (PLL) that acted as a biocompatible adhesive,^49^ preventing microgel movement during the measurement (Figure 2b). Surface treatment with PLL was crucial, providing mechanical stability during nanoindentation. The absence of the microgel adhesive layer caused microgels to slip off the surface during indentation, preventing the acquisition of reliable indentation curves with a linear response (Figure S5). Moreover, the concentration of PLL for this process needed to be carefully adjusted, given that the cationic polymer has been used as a secondary cross-linking agent to strengthen the integrity of the hydrogel network in cell-carrying microgels.^14^ Indeed, high concentrations of PLL beyond 0.5 mg/mL induced secondary cross-linking in M2-type microgels, permanently altering microgel size and density (Figure S6). Insufficient removal of PLL following the coating step similarly caused a clearly visible difference in the microgel size (Figure S6). On the other hand, PLL concentrations less than 0.1 mg/mL were insufficient in adhering microgels to the well plate surface, leading to microgel movement during the measurement (Video S1). Indentation measurements conducted this way either failed during the approaching step or resulted in incomplete force-strain curves. The optimal working concentration for PLL was identified as 0.1 mg/mL. In addition, complete removal of PLL by drying the substrate over a hot plate was necessary to prevent undesirable secondary cross-linking. Measurements were then conducted in deionized water to establish a baseline of the mechanical properties, free from external ions that might affect the cross-linking. Starting at the pole of the microgel, we mapped its curved surface in 1-μm steps (Video S2). While the microgel exhibited local deformation during the indentation process, its overall size stayed constant throughout the measurements, evidenced by in situ fluorescence imaging (Video S2). Within the first 5 μm from the microgel pole, the measured average Young’s modulus stayed constant at 5.4 ± 0.08 kPa (Figure S7). Beyond this region, a noticeable drop in stiffness was observed, likely due to incomplete indentation by the spherical tip. At these areas, the round geometry of the tip resulted in only partial engagement with the microgel surface. To ensure consistency, all subsequent measurements were carefully restricted to the pole region of the microgel.

Considering the potential influence of probe size and stiffness, we performed measurements using cantilevers with varying tip sizes (3 and 11 μm) and stiffness values (0.5 and 0.025 N/m). All probes revealed similar results with minor deviations of 0.3 kPa (Figure S8). Softer probes (∼0.02 N/m) were more susceptible to noise and thus harder to calibrate; we therefore continued the measurements with the stiffer probes. These tests adhered to manufacturer recommendations, maintaining indentation depths below 16% of the tip radius and within 5–10% of the sample thickness.^50^

Having developed a robust indentation method, we next evaluated the reliability of our measurement approach. For this purpose, macroscale alginate gels were prepared using RhodamineB-labeled alginate at a final concentration of 1 wt % and ionically cross-linking the prepolymer mixture via the dissolution of CaCO_3_ nanoparticles, following the formulation for M1-type microgels. In contrast to the microfluidic process, the acid glucono-δ-lactone (GDL) was included in the prepolymer mixture at 200 mg/mL concentration prior to casting the alginate macrogels for efficient cross-linking. Failure to include GDL in the prepolymer mixture prior to macrogel casting resulted in poorly cross-linked alginate gels, indicating the necessity for an actively initiated cross-linker dissolution process for efficient cross-linking at the macroscale (Figure S9). Alginate macrogels prepared via GDL were then characterized using a commercial shear rheometer in deionized water, which revealed storage moduli of 3.5 ± 0.4 kPa at 21.8 mM Ca^2+^ and 7.0 ± 0.6 kPa at 40.5 mM Ca^2+^, respectively (Figure S9). These values correspond to Young’s moduli of 10.7 ± 1.0 and 21.3 ± 2.1 kPa for 21.8 mM Ca^2+^ and 40.5 mM Ca^2+^, respectively. These measurements were in complete agreement with those carried out using nanoindentation on corresponding microgels. We recorded Young’s moduli of 11.8 ± 1.4 and 23.3 ± 1.3 kPa in deionized water for M1-type microgels fabricated using 21.8 and 40.5 mM Ca^2+^, respectively (Figure S10). Both micro- and macrogels exhibited a 50% increase in average stiffness with increased calcium cross-linker concentration. Taken together, these results proved the measurement reliability of our nanoindentation approach.

For further measurements, M2-type microgels were exclusively used, as they contain the modified alginate with an RGD integrin-binding peptide for cell adhesion and gold nanorods, creating an active microgel system that is more suitable in vitro culture conditions, in contrast to the static M1-type microgels. We investigated the influence of microgel size and cross-linker concentration on the Young’s moduli of M2-type microgels. The average stiffness of microgels produced at 21.8 mM of Ca^2+^ concentration was measured to be 8.4 ± 1.0 and 9.8 ± 0.8 kPa, corresponding to microgel sizes of 29.6 ± 0.9 and 36.3 ± 1.3 μm (Figure 2c). Similar observations were made with M2-type microgels fabricated at 40.5 mM Ca^2+^ concentration, where the microgel size did not influence microgel stiffness. In contrast, comparing the different cross-linker concentrations for M2-type microgels within the same size range revealed a drastic change in the microgel stiffness (Figure 2c). As expected, the average Young’s moduli of M2-type microgels produced at 40.5 mM Ca^2+^ was increased drastically by over 45%, reaching 14.4 ± 1.3 kPa (20-μm channel) and 14.5 ± 1.0 kPa (30-μm channel). Our observations match well with previous reports on macroscale alginate gels with similar composition, exhibiting stiffness within a range of 5–65 kPa.^51−54^

We briefly evaluated the possible influence of inorganic nanoparticles on the overall stiffness of the microgels. Incorporating Au nanoparticles into the microgels significantly affects their stiffness, as determined by a Wilcoxon signed-rank test (*P* < 0.001, ***, Figure S11). When measured in bead buffer, the mechanical properties of M2-type microgels without gold nanorods (0.6 ± 0.1 Pa) indicate a stiffer network than M2-type microgels with gold nanorods (0.5 ± 0.1 Pa). Although the reduction is subtle, this reduction in stiffness indicates that functionalization with gold nanorods introduces minor modifications to the ionic cross-linking network.

### Presence of CaCl_2_ and NaCl in Measurement Medium and Its Effect on Young’s Moduli of Alginate Microgels

**Figure 3:**
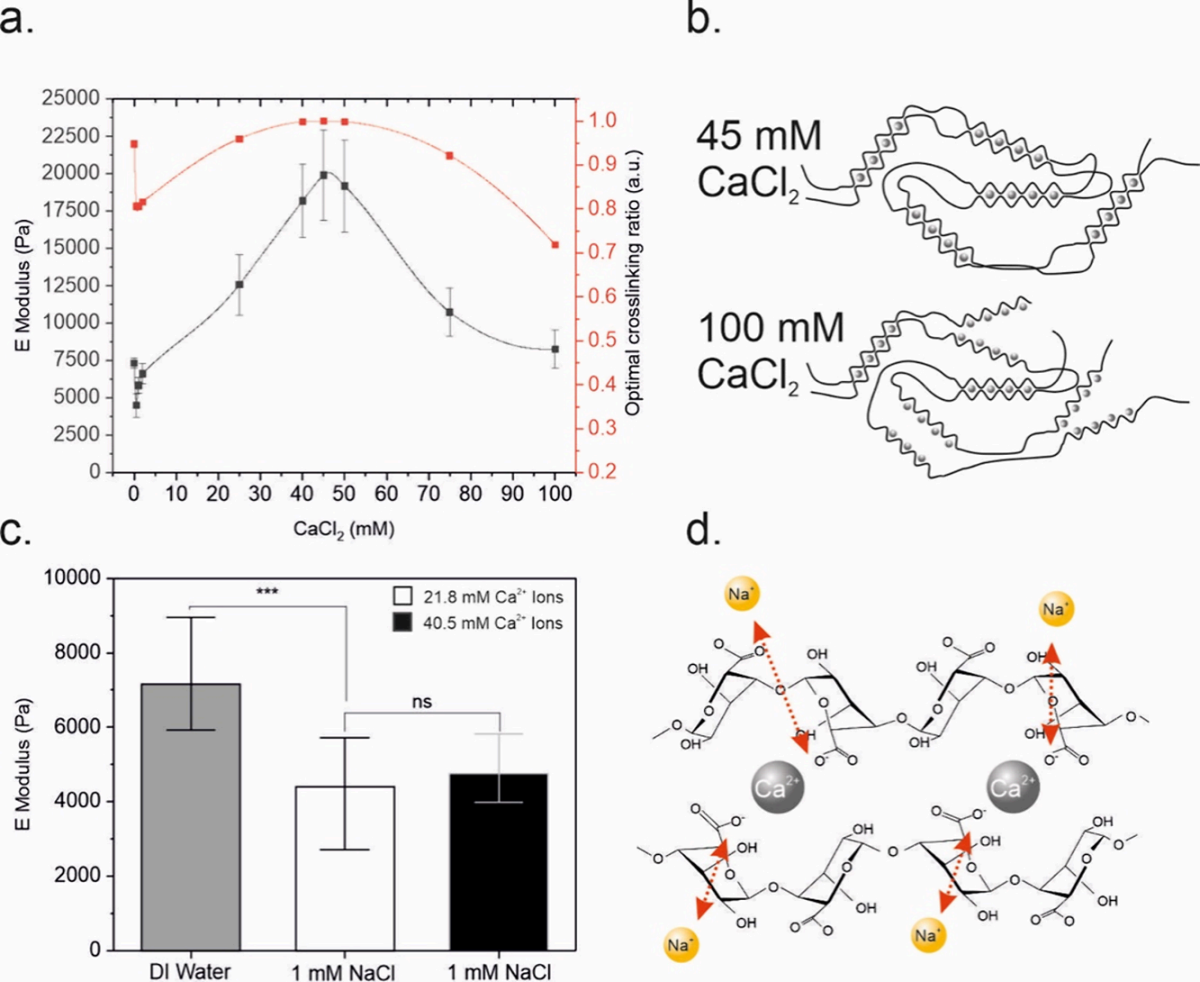
Nanoindentation measurements and schematic models of M2-type alginate microgels, in CaCl_2_ and NaCl environments. (a) Normalized, calculated optimal CaCl_2_ cross-linking ratio shown in red, with the highest theoretical cross-linking at 45 mM CaCl_2_. Nanoindentation measurements of M2-type microgels (fabricated at 21.8 mM Ca^2+^ concentration) are plotted in black and tested at CaCl_2_ concentrations of 0, 0.5, 1, 2, 25, 40, 45, 50, 75, and 100 mM. (b) Schematic egg-box model depicting ionically cross-linked alginate polymers. Full utilization of binding sites is illustrated at 50 mM CaCl_2_, with higher concentrations leading to overcrowding and subsequent network weakening. (c) Nanoindentation measurements of M2-type microgels, produced at 21.8 and 40.5 mM Ca^2+^ concentrations, in deionized water and 1 mM NaCl show significant softening of the microgels with NaCl (*n* = 15 per condition, ***P < 0.001, one-way ANOVA). (d) Schematic egg-box model illustrating chemical binding competition between Na^+^ and Ca^2+^ ions, with network depolymerization at elevated Na^+^ concentrations.

Calcium chloride (CaCl_2_) has been an essential source for maintaining and storing ionically cross-linked alginate microgels.^47,55^ Moreover, CaCl_2_ and NaCl are basic components in cell culture media for various mammalian cell types, such as Dulbecco’s modified Eagle’s medium (DMEM). We conjectured that it was essential to understand how varying concentrations of CaCl_2_ affect microgel stiffness, considering the differences in the concentration of CaCl_2_ across different cell culture media tailored to different cell types. We therefore investigated changes in the mechanical properties of M2-type microgels under different concentrations of CaCl_2_, particularly focusing on the strengthening effects of calcium ions on the alginate matrix. The M2-type microgels were specifically selected due to their integration of the RGD sequence, which facilitates cell adhesion via integrin binding, as well as nanoparticles embedded for laser actuation. These nanoparticles allow precise control over thermal and mechanical stimuli in mechanobiology studies, making the M2 microgel system ideal for *in vitro* cell culture applications where both mechanical modulation and bioactivity are essential.^9,41^ We hypothesized that microgels with the highest Young’s modulus would be generated at a concentration of CaCl_2_, where the molar ratio of Ca^2+^ ions in solution to carboxyl groups of alginate participating in ionic cross-linking was 1:1. Alginate is a copolymer consisting of mannuronate (M) and guluronate (G) acid residues, where only the carboxyl groups in the G blocks are accepted to participate in ionic cross-linking.^17,53,54^ The alginate used in this study had an M-to-G block ratio of 0.65, meaning that 39.4% of the monomer units are M-blocks and 60.6% are guluronic acid G-blocks.^56^ Cross-linking occurs specifically through the G-blocks, as calcium ions bind to the carboxyl groups on these blocks, forming ionic bridges. Importantly, every monomer unit in alginate, whether an M-block or a G-block, contains one carboxyl group. To calculate the optimal CaCl_2_ concentration for cross-linking, we considered the total concentration of carboxyl groups (*C*_Carboxyl_) in the alginate solution, using eq 3,
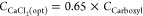
3where *C*_CaCl_2_(opt)_ is the optimal CaCl_2_ concentration in the aqueous phase, 0.65 is the target molar ratio of Ca^2+^ ions to available carboxyl groups, and *C*_Carboxyl_ is the concentration of carboxyl groups in the alginate solution. Given that *C*_Carboxyl_ in our microgel fabrication is 0.0124 mmol, *C*_CaCl_2_(opt)_ was calculated to be 0.008 mmol. The substance amount was adjusted to the volume of the alginate solution (1 wt % of 120 μL) and calcium-rich buffer was used to reconstitute the alginate, resulting in a CaCl_2_ concentration of 45 mM in the aqueous phase. This concentration achieved the ideal ratio of Ca^2+^ ions to carboxyl groups in the alginate, providing maximum ionic cross-linking and optimal microgel stiffness. As shown in Figure 3a, this optimal ratio of 0.65 was normalized to 1.0 on the graph, represented by a red line. Here, we assumed that the microgels would preserve the concentration of Ca^2+^ inside the hydrogel network. However, in practice, we expected a softening effect due to the diffusion of ions. Moving left or right from this optimal concentration (45 mM CaCl_2_) resulted in suboptimal cross-linking, as either lower or higher Ca^2+^ concentrations reduce the stiffness of the microgels. Excessive Ca^2+^ ions would alter the hydrogel’s mechanical behavior, as higher ion concentrations oversaturate the available carboxyl binding sites within the alginate network. This phenomenon was reported to lead to decreased elongation at break and lower swelling capacity, weakening the macrogel structure and reducing mechanical strength.^57^

We tested M2-type microgels (21.8 mM of Ca^2+^) using *in situ* nanoindentation and observed that in deionized water (0 mM of CaCl_2_), the average Young’s modulus was 7.3 ± 0.3 kPa (Figure 3a). When a small amount of CaCl_2_ (0.5 mM) was added to the medium, microgel stiffness decreased by 39% to 4.5 ± 0.8 kPa. Introducing Ca^2+^ ions at low concentrations in the aqueous media may have led to partial dissociation of the existing bonds, due to the dynamic nature of ionic cross-linking in alginate networks. However, as CaCl_2_ concentration in the media was increased to 1 and 2 mM, microgel stiffness was partially recovered, reaching average Young’s moduli of 5.9 ± 0.5 and 6.6 ± 0.7 kPa, respectively. Further increasing the CaCl_2_ concentration to 25 mM in media caused a drastic stiffening effect, increasing the average Young’s modulus by 90% to 12.6 ± 2.0 kPa compared to the baseline value. Microgel stiffness increased progressively with higher CaCl_2_ concentrations, reaching an average Young’s modulus of 18.2 ± 2.5 kPa at 40 mM, 19.9 ± 3.0 kPa at 45 mM, and 19.2 ± 4.1 kPa at 50 mM (Figure 3a). The highest stiffness value was observed at 45 mM, reflecting a 167% increase compared with lower concentrations. These results suggest that optimal cross-linking occurred at 45 mM CaCl_2_, aligning closely with our theoretical predictions (Figure 3a, red line). We additionally investigated changes in the stiffness of M2-type microgels initially cross-linked at 40.5 mM Ca^2+^, in the presence of 40 mM CaCl_2_. The Young’s modulus of 40.5 mM Ca^2+^ cross-linked microgels was 18.7 ± 3.7 kPa, closely aligning with the stiffness observed for 21.8 mM Ca^2+^ cross-linked microgels in 40 mM CaCl_2_ (18.2 ± 2.5 kPa). The results suggest that independent of starting calcium cross-linker conditions, microgels reach the same equilibrium cross-linking in the presence of high amounts of CaCl_2_. Interestingly, measurements on M2-type microgels revealed a softer network compared to what we initially estimated. This result was likely due to the diffusion of some Ca^2+^ ions from the network into aqueous media. As expected from theoretical calculations, increasing CaCl_2_ concentration in media beyond the optimal cross-linking point to 75 mM led to a large decrease in microgel stiffness (10.7 ± 1.6 kPa). This trend continued at 100 mM CaCl_2_, where the average Young’s modulus of M2-type microgels was measured to be 8.3 ± 1.3 kPa. These results show that overcrowding of binding sites disrupted optimal cross-linking (Figure 3b).

Next, we investigated the influence of sodium chloride (NaCl), which acts as a chelating agent on the ionic bonds within alginate networks.^58,59^ Using the same setup and methodology as in the previous experiments, we examined how varying concentrations of NaCl affected the mechanical properties of M2-type microgels. As expected, the introduction of 1 mM NaCl in the aqueous media led to a significant softening of the microgels (Figure 3c), which exhibited an average Young’s modulus of 4.3 ± 0.9 kPa as opposed to measurements in deionized water (7.2 ± 0.9 kPa). We did not observe any significant difference in Young’s moduli of M2-type microgels fabricated at 21.8 and 40.5 mM Ca^2+^ in the presence of 1 mM NaCl (Figure 3c). Increasing the NaCl concentration to 10 mM led to a loss in the structural integrity of microgels, which were ultimately completely dissolved at 20 mM (Video S3). These observations prove the competition between Na^+^ and Ca^2+^ ions for binding sites within the alginate network and the chelating effect of free Na^+^ in the aqueous environment causing network dissociation (Figure 3d). At concentrations above 10 mM, Na^+^ ions likely outcompete Ca^2+^ ions for binding sites within the alginate matrix, effectively disrupting the cross-linked structure that provides mechanical stability.

Interestingly, the dissociation behavior of macrogels under similar conditions displays both similarities and differences in time scale and concentration sensitivity. Macroscopic alginate gels exposed to physiological levels of NaCl (0.15 M) showed significant reductions in mechanical properties within 15 h, with compressive modulus dropping by 63% and shear modulus by 84% according to previous reports.^60^ Beyond this time scale, no further degradation was observed, suggesting a stabilization of the dissociation process over approximately 1 week.^60^ Comparatively, in microgels, the softening and dissolution occur at lower NaCl concentrations and on a shorter time scale, with complete dissociation observed at only 20 mM NaCl. It suggests that microgels’ higher surface area-to-volume ratio facilitates faster and more complete ionic exchange, making the structure more susceptible to disruption. Furthermore, while macrogels maintain some degree of structural integrity despite significant modulus reduction at physiological NaCl concentrations, microgels are completely destabilized at much lower thresholds.

### Synergistic Effects of CaCl_2_ and NaCl on Microgel Stiffness

To fully understand the behavior of microgels in complex environments, we conjectured that it was crucial to consider the combined effects of CaCl_2_ and NaCl on microgel stiffness because these salts often coexist in physiological conditions. The size of the M2-type microgels was first analyzed across different CaCl_2_ concentrations and varying levels of NaCl. The average microgel size decreased slightly to 32.3 ± 1.2 μm in the presence of low amounts of CaCl_2_ (2 mM), compared to a starting size of 36.3 ± 1.3 μm in deionized water (Table 1). Introducing NaCl under these conditions led to minor swelling, with the diameter rising to 33.3 ± 1.0 μm at 1 mM NaCl and reaching 34.6 ± 1.8 μm at 10 mM NaCl in the presence of 2 mM CaCl_2_. However, at 100 mM NaCl, the microgels expanded to an average size of 36.1 ± 1.0 μm, suggesting that high NaCl concentrations promote swelling, potentially due to osmotic effects or competitive binding with Ca^2+^. Removing NaCl from the aqueous media completely while increasing the CaCl_2_ concentration to 50 mM led to a substantial decrease in the microgel size (28.9 ± 1.0 μm). Introducing NaCl into the mixture at low concentrations (1 mM) caused an increase in microgel size reaching 33.3 ± 2.3 μm. From this point onward, microgel size remained unchanged even at much higher NaCl concentrations up to 100 mM. Our findings show that the influence of NaCl on microgel swelling was effectively moderated by the presence of CaCl_2_. The swelling behavior caused by NaCl in solution was inhibited at 100 mM CaCl_2_ concentration, where the microgel size remained constant within a range of 1–100 mM NaCl concentration (Table 1). The presence of large amounts of free Ca^2+^ in solution counteracts the swelling effect caused by Na^+^ presence, likely due to increased cross-linking density that maintains the microgel structure despite the presence of Na^+^ ions.

**Table 1:** M2-Microgel Stiffness and Size at Different NaCl/CaCl_2_ Concentrations

NaCl and CaCl concentrations	Youngs modulus (kPa)	stiffness trend	number of measurements	microgel size (um)	*N* size analysis
deionized water	7.3 ± 0.3	baseline	14	36.3 ± 1.3	100
0 mM NaCl/2 mM CaCl_2_	6.6 ± 0.7	–	22	32.3 ± 1.2	495
1 mM NaCl/2 mM CaCl_2_	8.7 ± 2.3	+	22	33.3 ± 1.0	517
10 mM NaCl/2 mM CaCl_2_	5.5 ± 0.9	––	16	34.6 ± 1.8	487
100 mM NaCl/2 mM CaCl_2_	0.6 ± 0.3	–––	15	36.1 ± 1.0	508
0 mM NaCl/50 mM CaCl_2_	19.2 ± 4.1	+++	16	28.9 ± 1.0	395
1 mM NaCl/50 mM CaCl_2_	14.4 ± 6.6	++	18	33.3 ± 2.3	429
10 mM NaCl/50 mM CaCl_2_	6.5 ± 0.7	–	14	32.9 ± 2.4	479
100 mM NaCl/50 mM CaCl_2_	11.1 ± 3.8	+	17	33.9 ± 2.0	342
0 mM NaCl/100 mM CaCl_2_	8.3 ± 1.3	+	11	28.9 ± 0.9	258
1 mM NaCl/100 mM CaCl_2_	8.4 ± 6.8	+	33	31.2 ± 1.5	849
10 mM NaCl/100 mM CaCl_2_	8.6 ± 0.9	+	21	29.0 ± 0.9	620
100 mM NaCl/100 mM CaCl_2_	16.9 ± 3.4	+++	37	29.2 ± 0.9	883

Having evaluated the morphological changes induced by ionic species in media, we next investigated the corresponding changes in the microgel stiffness (Figure S12). The average Young’s modulus of M2-type microgels was reduced to 6.6 ± 0.7 kPa in the presence of 2 mM CaCl_2_, which was increased to 8.7 ± 2.3 kPa with the addition of 1 mM NaCl (***P < 0.001). However, as NaCl concentration in the aqueous media was increased to 10 mM, microgel stiffness decreased to 5.5 ± 0.9 kPa (***P < 0.001). This trend was significantly enhanced at 100 mM NaCl, causing a drastic softening effect with the average Young’s modulus of microgels measured as 0.6 ± 0.3 kPa (***P < 0.001). These results clearly demonstrate the disruptive effect of Na^+^ on the ionically cross-linked alginate network, corroborated by the increase in microgel size under the same conditions. However, in the absence of NaCl and in the presence of 50 mM CaCl_2_, the microgel stiffness reached its highest value of 19.2 ± 4.1 kPa. At 50 mM CaCl_2_, a stepwise reduction in microgel stiffness was observed with a gradual increase of NaCl concentration from 14.4 ± 6.6 kPa at 1 mM NaCl (**P < 0.01) to a lower value of 6.5 ± 0.7 kPa at 10 mM NaCl (***P < 0.001). Interestingly, further increasing the NaCl concentration of the surrounding media to 100 mM led to an increase in microgel stiffness (11.1 ± 3.8 kPa), compared to 10 mM (*P* > 0.05, ns). From these results, it is evident that high concentrations of CaCl_2_ provide a means to counteract the drastic softening effect caused by free Na^+^ ions in solution. Stiffness measurements conducted on the same microgels in the presence of 100 mM CaCl_2_ showed similar results (Table 1). At 100 mM CaCl_2_, microgels exhibited Young’s moduli of about 8 kPa with increasing concentrations of NaCl up to 10 mM (*P* > 0.05, ns). The significant difference in microgel stiffness from 50 mM (19.2 ± 4.1) to 100 mM (8.3 ± 1.3) CaCl_2_ was likely due to overcrowding of binding sites (***P < 0.001). This finding supports our claim of 50 mM CaCl_2_ being the optimal cross-linking concentration, taken together with the constant microgel size under the two conditions. On the other hand, the introduction of high concentrations of NaCl (100 mM) likely inhibits overcrowding at the binding sites and enables more effective cross-linking, evidenced by the recovery of microgel stiffness (16.9 ± 3.4 kPa) to almost its highest value measured at 50 mM CaCl_2_ (19.2 ± 4.1 kPa) (***P < 0.001).

**Figure 4:**
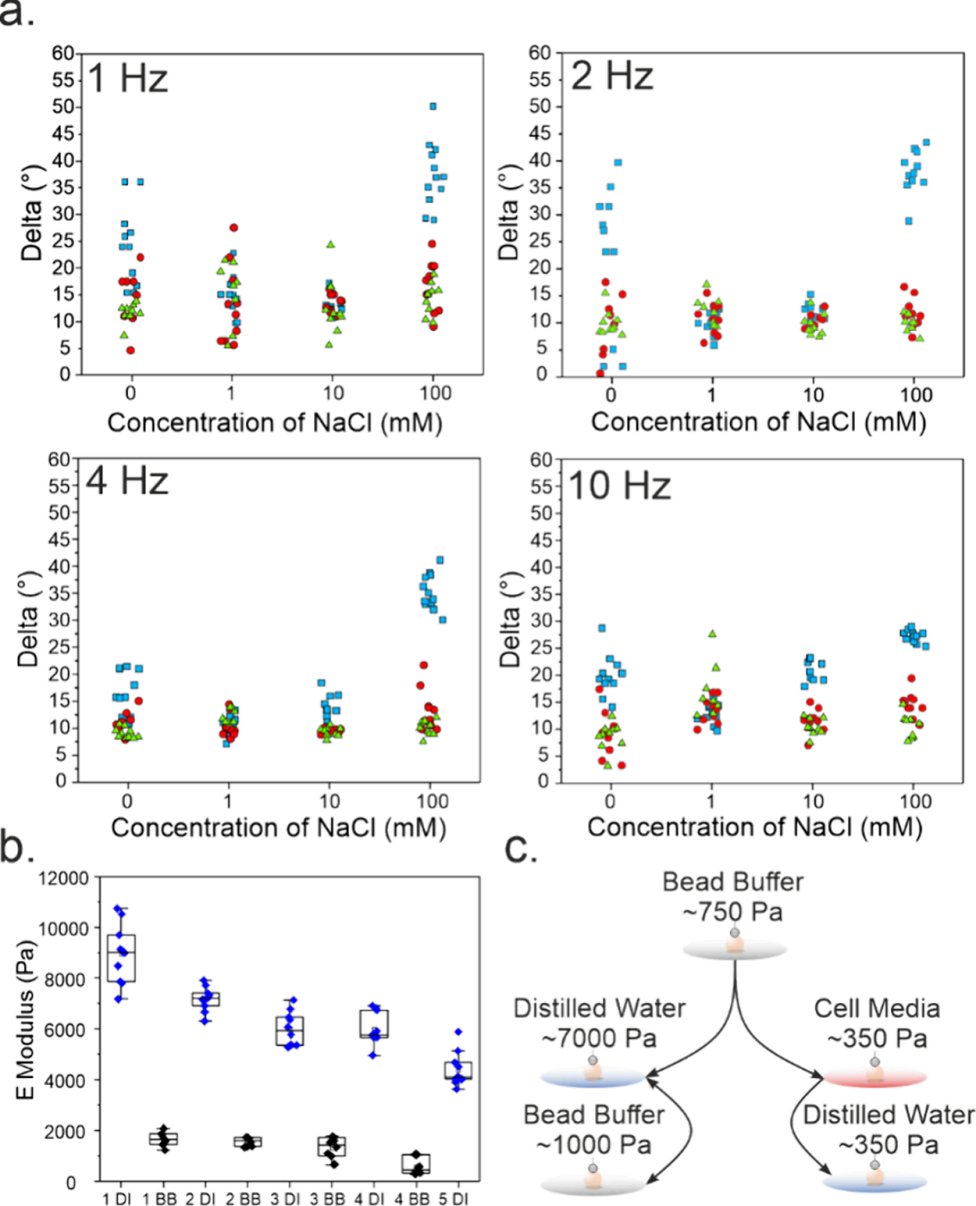
Viscoelastic analysis of M2-type alginate microgels. (a) Phase angle measurements for 21.8 mM Ca^2+^ M2-type microgels across varying NaCl concentrations (0, 1, 10, and 100 mM) and CaCl_2_ levels (blue squares: 2 mM CaCl_2_; red circles: 50 mM CaCl_2_; green triangles: 100 mM CaCl_2_) at frequencies of 1, 2, 4, and 10 Hz. (b) Young’s modulus measurements of 21.8 mM Ca^2+^ M2-type microgels in deionized water (DI) and bead buffer (BB), repeated over five cycles. (c) Schematic representation of reversible stiffness modulation in microgels for deionized water and bead buffer and permanent changes in cell media.

The presence of free Ca^2+^ and Na^+^ in the surrounding media of microgels similarly influenced the viscous behavior of the microgels. Characterization of the viscoelastic properties of M2-type microgels via DMA revealed viscous behavior at low Ca^2+^ (2 mM) concentration in the presence of high amounts of free Na^+^ in solution, evidenced by the drastic increase in the phase angle of the microgels reaching 38.1° at a 2 Hz oscillation frequency (Figure S13). As expected, microgels exhibited an elastic response at CaCl_2_ concentrations above 50 mM, as indicated by phase angles ranging from 11.6° (50 mM CaCl_2_) to 16.5° (100 mM CaCl_2_) measured at 1 Hz (Figure 4a).

### Impact of Storage Liquids and Cell Culture Media on Microgel Stiffness

We next investigated microgel stiffness in a range of different buffer solutions and cell culture media, considering the practical applications of alginate microgels. For this purpose, we first characterized the Young’s moduli of M2-type microgels in two buffer solutions we regularly use for long-term storage of alginate microgels, namely, HEPES buffer and bead buffer. While the average Young’s modulus of microgels in HEPES buffer solution was only slightly reduced compared to those in deionized water (Table 2), microgels were drastically softened in bead buffer, which consists of 130 mM NaCl, 2 mM CaCl_2_, and 25 mM HEPES. These results were in agreement with previously published work on alginate microgels, which reported average Young’s moduli within a range of 1–3 kPa, measured by AFM.^14,61^ It is important to point out that differences in the molecular weight of alginate, cross-linker type, and concentration and the presence of different functional moieties on alginate chains directly contribute to the measurement results. These differences, in turn, emphasize the importance of characterization methods tailored to microgels and their individual implementation. In pure HEPES buffer at the same concentration (25 mM) microgel stiffness was measured to be 6.2 ± 1.3, which decreased to 0.7 ± 0.1 kPa in the presence of high amounts of Na^+^ and low amounts of Ca^2+^ as expected from previously discussed measurements. The average stiffness of microgels was further reduced to a value of 0.2 ± 0.1 kPa in the presence of DMEM, which is used to culture a wide range of mammalian cells. This substantial decrease is likely caused by the higher ionic complexity of DMEM, which includes 2 mM CaCl_2_, 1 mM MgSO_4_, 5.4 mM KCl, and 110 mM NaCl, along with additional buffering agents, vitamins, and amino acids. The presence of all of these different chemical species disrupts cross-linking of the alginate network, evidenced by the decrease in stiffness and increase in microgel size.^62^ The inclusion of serum proteins, antibiotics, and buffer agents did not necessarily reverse the softening effect of DMEM (Table 2). Moreover, microgel stiffness further decreased when DMEM was exchanged with other kinds of cell culture media agents. In the presence of DMEM/F-12, RPMI 1640, and mTeSR Plus, microgel stiffness dropped below 0.1 kPa, below the resolution limit of the nanoindenter. A swelling effect was simultaneously observed, especially in the presence of RPMI 1640, likely due to its high osmotic and pH content, which has been reported to promote water uptake and weakening in ionic cross-links.^58,63,64^ Identifying the drastic softening effect induced by storage and cell culture media is crucial, because of the large range of Young’s moduli which has been shown to influence cell behavior.^65,66^ These results highlight the importance of choosing the correct media for characterizing the mechanical properties of ionically cross-linked alginate microgels. Otherwise, differences in the composition of liquid media used during fabrication, storage, and practical applications of alginate microgels could lead to unreliable measurements.

**Table 2:** M2-Microgel Stiffness in Deionized Water, Storage Buffer, and Cell Media

media	*E* (kPa) (*N* ≥ 10)	diameter (μm) (*N* ≥ 381)	measurable	pH	reversibility
deionized water	7.2 ± 0.9	36.0 ± 0.7	yes	7	yes
HEPES Buffer (25 mM)	6.2 ± 1.3	34.9 ± 1.0	yes	6.5	
d-glucose (25 mM)	5.5 ± 0.8	33.5 ± 0.8	yes	6.5	
bead buffer	0.7 ± 0.1	34.7 ± 1.0	yes	6.5	yes
DMEM + FBS (10%) + P/S (1%)	0.4 ± 0.1	37.0 ± 1.0	yes	9	
DMEM	0.2 ± 0.1	38.4 ± 1.4	yes	9	no
DMEM w/o NaHCO_3_	0.4 ± 0.1	36.1 ± 0.9	yes	6	
DMEM + HEPES	0.3 ± 0.1	36.0 ± 1.0	yes	7.5	
DMEM + HEPES + CaCO_3_	0.3 ± 0.1	36.1 ± 0.9	yes	8	
DMEM/F-12	<0.1	37.3 ± 0.7	no	7	
RPMI 1640	<0.1	44.9 ± 1.3	no	9	
mTeSR plus	<0.1	36.1 ± 1.3	no	8	

We next studied the reversibility of the observed changes in microgel stiffness. For this purpose, microgels treated in bead buffer were transferred to deionized water and measured following a 10 min incubation time (Figure 4b), which was repeated over 5 cycles. The average Young’s modulus of the microgels was increased as the microgels were transferred from bead buffer to deionized water within each cycle. However, comparing the initial and final stiffness values measured in deionized water indicated a reduction of about 55% in the microgel stiffness in an irreversible manner. Interestingly, the average stiffness of microgels treated only once with cell culture medium DMEM remained constant even after exposure to deionized water. These results suggest that serum proteins and complex media constituents cause lasting structural changes (Figure 4c), in line with similar reports on softening in serum-rich hydrogels.^67^

## Conclusions

Here, we investigated the mechanical properties of ionically cross-linked alginate microgels, by paying close attention to the presence of mono- and divalent ions in solution. We validated the chelating effect of NaCl on calcium-cross-linked alginate microgels and systematically evaluated changes in microgel stiffness *via* nanoindentation. The effect of cross-linker concentration, microgel size, and ionic composition of measurement medium on the average Young’s moduli of alginate microgels was characterized in detail, demonstrating the versatility of our nanoindentation-based measurement strategy. Overall, nanoindentation enabled robust and reliable mechanical characterization of microgels, in terms of stiffness and viscoelasticity, validated by rheology measurements on the corresponding macrogels. While suitable for most applications, the spatial resolution for nanoindentation is lower than that achieved with AFM, potentially limiting the detection of finer structural details. In addition, the nanoindentation setup that we used is currently incapable of maintaining the sterile conditions necessary for long-term cell studies. Addressing this particular challenge in the future will ensure the applicability of the measurement strategy to long-term biological experiments.

We summarize our results in a list of guidelines applicable to the mechanical characterization of ionically cross-linked alginate microgels using probe-based techniques.

- Choice of alginate and microgel composition: The properties of alginate and the formulation used to fabricate the microgels are important factors that can influence the mechanical properties of the resulting microgels. Here, we characterized the Young’s moduli of microgels fabricated with high molecular weight alginate (280 kDa) with a M-to-G-block ratio of 0.65, which was functionalized with biomolecules (e.g., biotin, RGD-peptides). Microgels produced at 21.8 mM Ca^2+^ concentration exhibited stiffness values of about 9 kPa, which increased to an approximate value of 14 kPa at 40.5 mM Ca^2+^. The introduction of inorganic nanomaterials in the microgels additionally influenced these values. However, microgel size did not influence the mechanical properties.
- Sample preparation prior to nanoindentation: Ensuring strong microgel adhesion to the measurement surface is crucial for successful nanoindentation. Functionalizing the substrate surface with PLL proved to be an effective strategy for ionically cross-linked alginate microgels used in this study, however, the concentration of PLL should not exceed 0.5 mg/L to prevent secondary cross-linking. We recommend using 0.1 mg/L and complete removal of excess PLL by drying prior to microgel adhesion.
- Choice of nanoindentation probe: This study used spherical probes with varying tip sizes (3 and 11 μm) and stiffness values (0.5 and 0.025 N/m) to evaluate their impact on measurement precision. While no changes in the measured stiffness were observed due to tip size, softer probes (0.025 N/m) introduced higher noise levels. Probes with a stiffness of 0.5 N/m provided the most consistent results at indentation depths of 1000 nm. We recommend selecting cantilevers with similar stiffness and spherical tips with smaller radii compared to the microgels to enable correct tip positioning.
- Ionic composition of aqueous microgel medium: The presence of CaCl_2_ promoted cross-linking, while NaCl induced disruption of ionic cross-links in alginate microgels. We found that the optimal cross-linking concentration was achieved at 45 mM CaCl_2_ leading to stiffness values of about ∼20 kPa, which was high enough to counteract the distruptive effects of NaCl within a range of 1–100 mM. However, low concentrations of CaCl_2_ and high concentrations of NaCl caused a drastic softening effect. Similar observations were made in different cell culture media, where alginate microgels exhibited stiffness values less than 1 kPa. Considering these findings, it is essential to correctly determine the measurement medium. We suggest conducting the measurements in media that correspond to the application of the microgels. Furthermore, the storage media for alginate microgels should be carefully selected, to avoid any temporal changes in microgel stiffness.

## Supporting Information Available

Undesirable microgel motion during indentation without PLL coating on the substrate (MPG)
Stable nanoindentation procedure in mapping mode over the microgel surface, enabled by secure attachment of microgel to the substrate surface (MP4)
Change in microgel integrity under increasing concentrations (1, 10, and 20 mM) of NaCl (MPG)
Viscoelastic moduli (1 Hz) of M1-type RhB alginate macrogel prepolymer mixture; microgels fabricated with 3.1 mM Ca^2+^ ions; microgels fabricated with varying flow speeds resulting in size differences; exemplary images of M2-type, gold nanoparticle carrying, alginate microgels made with 21.8–40.5 mM Ca^2+^ cross-linker concentrations; exemplary nanoindentation load curve; secondary cross-linking of microgels through high amounts of PLL; nanoindentation scan of a single microgel; comparison of different nanoindentation probes used to measure the same microgel; viscoelastic properties of 21.8 and 40.5 mM Ca^2+^ M1 alginate macrogels and the calculated Young’s moduli (*E*); microscope images and nanoindentation measurements of M1-type microgels; nanoindentation results of M2-type microgels with and without gold nanorods; comparison of Young’s moduli (*E*) of M2-type microgels under different NaCl and CaCl_2_ conditions; and exemplary nanoindentation load curve using DMA (PDF)

## Supplementary Material

am4c20952_si_001.mpg

am4c20952_si_002.mp4

am4c20952_si_003.mpg

## Abbreviations

BB: bead buffer
CaCO_3_: Calcium Carbonate
DMEM: Dulbecco’s Modified Eagle Medium
FBS: fetal bovine serum
P/S: penicillin/streptomycin
RhB: Rhodamine B
RPMI: Roswell Park Memorial Institute

## References

[ref1] Caldwell A. S.; Aguado B. A.; Anseth K. S. Designing Microgels for Cell Culture and Controlled Assembly of Tissue Microenvironments. Adv. Funct Mater. 2020, 30 (37), 1–15. 10.1002/adfm.201907670. PMC8026140 33841061

[ref2] Kamperman T.; Willemen N. G. A.; Kelder C.; Koerselman M.; Becker M.; Lins L.; Johnbosco C.; Karperien M.; Leijten J. Steering Stem Cell Fate within 3D Living Composite Tissues Using Stimuli-Responsive Cell-Adhesive Micromaterials. Advanced Science 2023, 10 (10), 1–15. 10.1002/advs.202205487. PMC10074101 36599686

[ref3] Dubay R.; Urban J. N.; Darling E. M. Single-Cell Microgels for Diagnostics and Therapeutics. Adv. Funct Mater. 2021, 31 (44), 1–28. 10.1002/adfm.202009946. PMC9629779 36329867

[ref4] Sivakumaran D.; Maitland D.; Hoare T. Injectable Microgel-Hydrogel Composites for Prolonged Small-Molecule Drug Delivery. Biomacromolecules 2011, 12 (11), 4112–4120. 10.1021/bm201170h. 22007750

[ref5] Sivakumaran D.; Maitland D.; Oszustowicz T.; Hoare T. Tuning Drug Release from Smart Microgel-Hydrogel Composites via Crosslinking. J. Colloid Interface Sci. 2013, 392 (1), 422–430. 10.1016/j.jcis.2012.07.096. 23137903

[ref6] Jeon S.; Kim S.; Ha S.; Lee S.; Kim E.; Kim S. Y.; Park S. H.; Jeon J. H.; Kim S. W.; Moon C.; Nelson B. J.; Kim J. Y.; Yu S. W.; Choi H. Magnetically Actuated Microrobots as a Platform for Stem Cell Transplantation. Sci. Robot 2019, 4 (30), 1–11. 10.1126/scirobotics.aav4317. 33137727

[ref7] Zimmermann H.; Zimmermann D.; Reuss R.; Feilen P. J.; Manz B.; Katsen A.; Weber M.; Ihmig F. R.; Ehrhart F.; Geßner P.; Behringer M.; Steinbach A.; Wegner L. H.; Sukhorukov V. L.; Vásquez J. A.; Schneider S.; Weber M. M.; Volke F.; Wolf R.; Zimmermann U. Towards a Medically Approved Technology for Alginate-Based Microcapsules Allowing Long-Term Immunoisolated Transplantation. J. Mater. Sci. Mater. Med. 2005, 16 (6), 491–501. 10.1007/s10856-005-0523-2. 15928863

[ref8] İyisan N.; Hausdörfer O.; Wang C.; Hiendlmeier L.; Harder P.; Wolfrum B.; Özkale B. Mechanoactivation of Single Stem Cells in Microgels Using a 3D-Printed Stimulation Device. Small Methods 2024, 8, 2400272 10.1002/smtd.202400272. 39011729 PMC11672187

[ref9] Özkale B.; Lou J.; Özelçi E.; Elosegui-Artola A.; Tringides C. M.; Mao A. S.; Sakar M. S.; Mooney D. J. Actuated 3D Microgels for Single Cell Mechanobiology. Lab Chip 2022, 22, 1962–1970. 10.1039/D2LC00203E. 35437554 PMC10116575

[ref10] Qazi T. H.; Burdick J. A. Granular Hydrogels for Endogenous Tissue Repair. Biomater. Biosyst. 2021, 1, 100008 10.1016/j.bbiosy.2021.100008. 36825161 PMC9934473

[ref11] Lowen J. M.; Bond G. C.; Griffin K. H.; Shimamoto N. K.; Thai V. L.; Leach J. K. Multisized Photoannealable Microgels Regulate Cell Spreading, Aggregation, and Macrophage Phenotype through Microporous Void Space. Adv. Healthc Mater. 2023, 12 (13), 1–14. 10.1002/adhm.202202239. PMC10198868 36719946

[ref12] Mohagheghian E.; Luo J.; Chen J.; Chaudhary G.; Chen J.; Sun J.; Ewoldt R. H.; Wang N. Quantifying Compressive Forces between Living Cell Layers and within Tissues Using Elastic Round Microgels. Nat. Commun. 2018, 9 (1), 1878 10.1038/s41467-018-04245-1. 29760452 PMC5951850

[ref13] Girardo S.; Träber N.; Wagner K.; Cojoc G.; Herold C.; Goswami R.; Schlüßler R.; Abuhattum S.; Taubenberger A.; Reichel F.; Mokbel D.; Herbig M.; Schürmann M.; Müller P.; Heida T.; Jacobi A.; Ulbricht E.; Thiele J.; Werner C.; Guck J. Standardized Microgel Beads as Elastic Cell Mechanical Probes. J. Mater. Chem. B 2018, 6 (39), 6245–6261. 10.1039/C8TB01421C. 32254615

[ref14] Mao A. S.; Shin J. W.; Utech S.; Wang H.; Uzun O.; Li W.; Cooper M.; Hu Y.; Zhang L.; Weitz D. A.; Mooney D. J. Deterministic Encapsulation of Single Cells in Thin Tunable Microgels for Niche Modelling and Therapeutic Delivery. Nat. Mater. 2017, 16 (2), 236–243. 10.1038/nmat4781. 27798621 PMC5372217

[ref15] Wong S. W.; Tamatam C. R.; Cho I. S.; Toth P. T.; Bargi R.; Belvitch P.; Lee J. C.; Rehman J.; Reddy S. P.; Shin J. W. Inhibition of Aberrant Tissue Remodelling by Mesenchymal Stromal Cells Singly Coated with Soft Gels Presenting Defined Chemomechanical Cues. Nat. Biomed Eng. 2022, 6 (1), 54–66. 10.1038/s41551-021-00740-x. 34083763 PMC8908879

[ref16] Mao A. S.; Özkale B.; Shah N. J.; Vining K. H.; Descombes T.; Zhang L.; Tringides C. M.; Wong S. W.; Shin J. W.; Scadden D. T.; Weitz D. A.; Mooney D. J. Programmable Microencapsulation for Enhanced Mesenchymal Stem Cell Persistence and Immunomodulation. Proc. Natl. Acad. Sci. U. S. A. 2019, 116 (31), 15392–15397. 10.1073/pnas.1819415116. 31311862 PMC6681761

[ref17] Lee K. Y.; Mooney D. J. Alginate: Properties and Biomedical Applications. In Progress in Polymer Science (Oxford); Elsevier Ltd, 2012; pp. 106–126. 10.1016/j.progpolymsci.2011.06.003 PMC3223967 22125349

[ref18] Utech S.; Prodanovic R.; Mao A. S.; Ostafe R.; Mooney D. J.; Weitz D. A. Microfluidic Generation of Monodisperse, Structurally Homogeneous Alginate Microgels for Cell Encapsulation and 3D Cell Culture. Adv. Healthc Mater. 2015, 4 (11), 1628–1633. 10.1002/adhm.201500021. 26039892 PMC4529809

[ref19] Benoit D. S. W.; Schwartz M. P.; Durney A. R.; Anseth K. S. Small Functional Groups for Controlled Differentiation of Hydrogel-Encapsulated Human Mesenchymal Stem Cells. Nat. Mater. 2008, 7 (10), 816–823. 10.1038/nmat2269. 18724374 PMC2929915

[ref20] Rowley J. A.; Madlambayan G.; Mooney D. J. Alginate Hydrogels as Synthetic Extracellular Matrix Materials. - 1999 - Rowley, Madlambayan, Mooney.Pdf. Biomaterials 1999, 20, 45–53. 10.1016/S0142-9612(98)00107-0. 9916770

[ref21] Augst A. D.; Kong H. J.; Mooney D. J. Alginate Hydrogels as Biomaterials. Macromol. Biosci 2006, 6 (8), 623–633. 10.1002/mabi.200600069. 16881042

[ref22] Chaudhuri O.; Gu L.; Klumpers D.; Darnell M.; Bencherif S. A.; Weaver J. C.; Huebsch N.; Lee H. P.; Lippens E.; Duda G. N.; Mooney D. J. Hydrogels with Tunable Stress Relaxation Regulate Stem Cell Fate and Activity. Nat. Mater. 2016, 15 (3), 326–334. 10.1038/nmat4489. 26618884 PMC4767627

[ref23] Thomas A.; Harding K. G.; Moore K. Alginates from Wound Dressings Activate Human Macrophages to Secrete Tumour Necrosis Factor-α. Biomaterials 2000, 21 (17), 1797–1802. 10.1016/S0142-9612(00)00072-7. 10905462

[ref24] Branco da Cunha C.; Klumpers D. D.; Li W. A.; Koshy S. T.; Weaver J. C.; Chaudhuri O.; Granja P. L.; Mooney D. J. Influence of the Stiffness of Three-Dimensional Alginate/Collagen-I Interpenetrating Networks on Fibroblast Biology. Biomaterials 2014, 35 (32), 8927–8936. 10.1016/j.biomaterials.2014.06.047. 25047628

[ref25] Huebsch N.; Arany P. R.; Mao A. S.; Shvartsman D.; Ali O. A.; Bencherif S. A.; Rivera-Feliciano J.; Mooney D. J. Harnessing Traction-Mediated Manipulation of the Cell/Matrix Interface to Control Stem-Cell Fate. Nat. Mater. 2010, 9 (6), 518–526. 10.1038/nmat2732. 20418863 PMC2919753

[ref26] Liu C.; Wu Y.; Yang H.; Lu K.; Zhang H.; Wang Y.; Wang J.; Ruan L.; Shen Z.; Yu Q.; Zhang Y. An Injectable Alginate/Fibrin Hydrogel Encapsulated with Cardiomyocytes and VEGF for Myocardial Infarction Treatment. J. Mater. Sci. Technol. 2023, 143, 198–206. 10.1016/j.jmst.2022.11.002.

[ref27] Charbonier F.; Indana D.; Chaudhuri O. Tuning Viscoelasticity in Alginate Hydrogels for 3D Cell Culture Studies. Curr. Protoc 2021, 1 (5), 1–28. 10.1002/cpz1.124. PMC8171168 34000104

[ref28] Zhang K.; Yang Z.; Seitz M. P.; Jain E. Macroporous PEG-Alginate Hybrid Double-Network Cryogels with Tunable Degradation Rates Prepared via Radical-Free Crosslinking for Cartilage Tissue Engineering. ACS Appl. Bio Mater. 2024, 7, 5925 10.1021/acsabm.4c00091. PMC11409214 39135543

[ref29] Mo̷rch Ä. A.; Donati I.; Strand B. L.; Skja G. Effect of Ca 2+, Ba 2+, and Sr 2+ on Alginate Microbeads. Biomacromolecules 2006, 7, 1471–1480. 10.1021/bm060010d. 16677028

[ref30] Wang C.; Harder P.; İyisan N.; Li B.; Hiendlmeier L.; Wolfrum B.; Özkale B. A Multiscale Approach to Assess Thermomechanical Performance and Force Generation in Nanorobotic Microgels. Nanoscale 2024, 16 (10), 5222–5231. 10.1039/D3NR06485A. 38354060

[ref31] Webber R. E.; Shull K. R. Strain Dependence of the Viscoelastic Properties of Alginate Hydrogels. Macromolecules 2004, 37 (16), 6153–6160. 10.1021/ma049274n.

[ref32] Hashemnejad S. M.; Kundu S. Rheological Properties and Failure of Alginate Hydrogels with Ionic and Covalent Crosslinks. Soft Matter 2019, 15 (39), 7852–7862. 10.1039/C9SM01039D. 31531488

[ref33] Kong H. J.; Kaigler D.; Kim K.; Mooney D. J. Controlling Rigidity and Degradation of Alginate Hydrogels via Molecular Weight Distribution. Biomacromolecules 2004, 5 (5), 1720–1727. 10.1021/bm049879r. 15360280

[ref34] Hecht H.; Srebnik S. Structural Characterization of Sodium Alginate and Calcium Alginate. Biomacromolecules 2016, 17 (6), 2160–2167. 10.1021/acs.biomac.6b00378. 27177209

[ref35] Drury J. L.; Mooney D. J. Hydrogels for Tissue Engineering: Scaffold Design Variables and Applications. Biomaterials 2003, 24 (24), 4337–4351. 10.1016/S0142-9612(03)00340-5. 12922147

[ref36] Decho A. W. Imaging an Alginate Polymer Gel Matrix Using Atomic Force Microscopy. Carbohydr. Res. 1999, 315 (3–4), 330–333. 10.1016/S0008-6215(99)00006-3.

[ref37] Distler T.; Kretzschmar L.; Schneidereit D.; Girardo S.; Goswami R.; Friedrich O.; Detsch R.; Guck J.; Boccaccini A. R.; Budday S. Mechanical Properties of Cell- And Microgel Bead-Laden Oxidized Alginate-Gelatin Hydrogels. Biomater Sci. 2021, 9 (8), 3051–3068. 10.1039/D0BM02117B. 33666608

[ref38] Lemon W. C.; McDole K. Live-Cell Imaging in the Era of Too Many Microscopes. Curr. Opin Cell Biol. 2020, 66, 34–42. 10.1016/j.ceb.2020.04.008. 32470820

[ref39] Dufrêne Y. F.; Ando T.; Garcia R.; Alsteens D.; Martinez-Martin D.; Engel A.; Gerber C.; Müller D. J. Imaging Modes of Atomic Force Microscopy for Application in Molecular and Cell Biology. Nat. Nanotechnol 2017, 12 (4), 295–307. 10.1038/nnano.2017.45. 28383040

[ref40] Kollmannsberger P.; Fabry B. Linear and Nonlinear Rheology of Living Cells. Annu. Rev. Mater. Res. 2011, 41, 75–97. 10.1146/annurev-matsci-062910-100351.

[ref41] Harder P.; İyisan N.; Wang C.; Kohler F.; Neb I.; Lahm H.; Dreßen M.; Krane M.; Dietz H.; Özkale B. A Laser-Driven Microrobot for Thermal Stimulation of Single Cells. Adv. Healthc Mater. 2023, 12 (26), 1–11. 10.1002/adhm.202300904. PMC11468149 37229536

[ref42] Babu S.; Chen I.; Vedaraman S.; Gerardo-Nava J.; Licht C.; Kittel Y.; Haraszti T.; Di Russo J.; De Laporte L. How Do the Local Physical, Biochemical, and Mechanical Properties of an Injectable Synthetic Anisotropic Hydrogel Affect Oriented Nerve Growth?. Adv. Funct. Mater. 2022, 32 (50), 2202468 10.1002/adfm.202202468.

[ref43] Rommel D.; Mork M.; Vedaraman S.; Bastard C.; Guerzoni L. P. B.; Kittel Y.; Vinokur R.; Born N.; Haraszti T.; De Laporte L. Functionalized Microgel Rods Interlinked into Soft Macroporous Structures for 3D Cell Culture. Adv. Sci. 2022, 9 (10), 2103554 10.1002/advs.202103554. PMC8981485 35032119

[ref44] Krüger A. J. D.; Bakirman O.; Guerzoni L. P. B.; Jans A.; Gehlen D. B.; Rommel D.; Haraszti T.; Kuehne A. J. C.; De Laporte L. Compartmentalized Jet Polymerization as a High-Resolution Process to Continuously Produce Anisometric Microgel Rods with Adjustable Size and Stiffness. Adv. Mater. 2019, 31 (49), 1903668 10.1002/adma.201903668. 31621960

[ref45] Roberge C. L.; Kingsley D. M.; Cornely L. R.; Spain C. J.; Fortin A. G.; Corr D. T. Viscoelastic Properties of Bioprinted Alginate Microbeads Compared to Their Bulk Hydrogel Analogs. J. Biomech Eng. 2023, 145 (3), 1–11. 10.1115/1.4055757. PMC9791675 36149022

[ref46] Lai Y.; Hu Y. Probing the Swelling-Dependent Mechanical and Transport Properties of Polyacrylamide Hydrogels through AFM-Based Dynamic Nanoindentation. Soft Matter 2018, 14 (14), 2619–2627. 10.1039/C7SM02351K. 29577116

[ref47] Hu Y.; Mao A. S.; Desai R. M.; Wang H.; Weitz D. A.; Mooney D. J. Controlled Self-Assembly of Alginate Microgels by Rapidly Binding Molecule Pairs. Lab Chip 2017, 17 (14), 2481–2490. 10.1039/C7LC00500H. 28627581 PMC5559697

[ref48] Jia P.; Zhao X.; Liu Y.; Liu M.; Zhang Q.; Chen S.; Huang H.; Jia Y.; Chang Y.; Han Z.; Han Z. C.; Li Q.; Guo Z.; Li Z. The RGD-Modified Self-Assembling D-Form Peptide Hydrogel Enhances the Therapeutic Effects of Mesenchymal Stem Cells (MSC) for Hindlimb Ischemia by Promoting Angiogenesis. Chemical Engineering Journal 2022, 450 (P1), 138004 10.1016/j.cej.2022.138004.

[ref49] Khademhosseini A.; Suh K. Y.; Yang J. M.; Eng G.; Yeh J.; Levenberg S.; Langer R. Layer-by-Layer Deposition of Hyaluronic Acid and Poly-L-Lysine for Patterned Cell Co-Cultures. Biomaterials 2004, 25 (17), 3583–3592. 10.1016/j.biomaterials.2003.10.033. 15020132

[ref50] Lin D. C.; Shreiber D. I.; Dimitriadis E. K.; Horkay F. Spherical Indentation of Soft Matter beyond the Hertzian Regime: Numerical and Experimental Validation of Hyperelastic Models. Biomech Model Mechanobiol 2009, 8 (5), 345–358. 10.1007/s10237-008-0139-9. 18979205 PMC3615644

[ref51] Freeman F. E.; Kelly D. J. Tuning Alginate Bioink Stiffness and Composition for Controlled Growth Factor Delivery and to Spatially Direct MSC Fate within Bioprinted Tissues. Sci. Rep. 2017, 7 (1), 17042 10.1038/s41598-017-17286-1. 29213126 PMC5719090

[ref52] Tumarkin E.; Kumacheva E. Microfluidic Generation of Microgels from Synthetic and Natural Polymers. Chem. Soc. Rev. 2009, 38 (8), 2161–2168. 10.1039/b809915b. 19623340

[ref53] Kuo C. K.; Ma P. X. Ionically Crosslinked Alginate Hydrogels as Scaffolds for Tissue Engineering: Part 1. Structure, Gelation Rate and Mechanical Properties. Biomaterials 2001, 22 (6), 511–521. 10.1016/S0142-9612(00)00201-5. 11219714

[ref54] Mancini M.; Moresi M.; Rancini R. Mechanical Properties of Alginate Gels: Empirical Characterisation. J. Food Eng. 1999, 39 (4), 369–378. 10.1016/S0260-8774(99)00022-9.

[ref55] Drury J. L.; Dennis R. G.; Mooney D. J. The Tensile Properties of Alginate Hydrogels. Biomaterials 2004, 25 (16), 3187–3199. 10.1016/j.biomaterials.2003.10.002. 14980414

[ref56] Sakai S.; Ono T.; Ijima H.; Kawakami K. Permeability of Alginate/Sol-Gel Synthesized Aminopropyl-Silicate/Alginate Membrane Templated by Calcium-Alginate Gel. J. Membr. Sci. 2002, 205 (1–2), 183–189. 10.1016/S0376-7388(02)00093-5.

[ref57] Li J.; Wu Y.; He J.; Huang Y. A New Insight to the Effect of Calcium Concentration on Gelation Process and Physical Properties of Alginate Films. J. Mater. Sci. 2016, 51 (12), 5791–5801. 10.1007/s10853-016-9880-0.

[ref58] Matyash M.; Despang F.; Ikonomidou C.; Gelinsky M. Swelling and Mechanical Properties of Alginate Hydrogels with Respect to Promotion of Neural Growth. Tissue Eng. Part C Methods 2014, 20 (5), 401–411. 10.1089/ten.tec.2013.0252. 24044417

[ref59] Mikula K.; Skrzypczak D.; Ligas B.; Witek-Krowiak A. Preparation of Hydrogel Composites Using Ca2+ and Cu2+ Ions as Crosslinking Agents. SN Appl. Sci. 2019, 1 (6), 1–15. 10.1007/s42452-019-0657-3.

[ref60] LeRoux M. A.; Guilak F.; Setton L. A. Compressive and Shear Properties of Alginate Gel: Effects of Sodium Ions and Alginate Concentration. J. Biomed Mater. Res. 1999, 47 (1), 46–53. 10.1002/(SICI)1097-4636(199910)47:1<46::AID-JBM6>3.0.CO;2-N. 10400879

[ref61] Lou J.; Meyer C.; Vitner E. B.; Adu-Berchie K.; Dacus M. T.; Bovone G.; Chen A.; To T.; Weitz D. A.; Mooney D. J. Surface-Functionalized Microgels as Artificial Antigen-Presenting Cells to Regulate Expansion of T Cells. Adv. Mater. 2024, 36 (31), 1–13. 10.1002/adma.202309860. PMC11293993 38615189

[ref62] Echalier C.; Valot L.; Martinez J.; Mehdi A.; Subra G. Chemical Crosslinking Methods for Cell Encapsulation in Hydrogels. Mater. Today Commun. 2019, 20 (May), 100536 10.1016/j.mtcomm.2019.05.012.

[ref63] Alziyadi M. O.; Denton A. R. Osmotic Swelling Behavior of Surface-Charged Ionic Microgels. J. Chem. Phys. 2023, 159 (18), 184901 10.1063/5.0161027. 37942869

[ref64] Jing Z.; Dai X.; Xian X.; Du X.; Liao M.; Hong P.; Li Y. Tough, Stretchable and Compressive Alginate-Based Hydrogels Achieved by Non-Covalent Interactions. RSC Adv. 2020, 10 (40), 23592–23606. 10.1039/D0RA03733H. 35517309 PMC9054928

[ref65] Chen J.; Irianto J.; Inamdar S.; Pravincumar P.; Lee D. A.; Bader D. L.; Knight M. M. Cell Mechanics, Structure, and Function Are Regulated by the Stiffness of the Three-Dimensional Microenvironment. Biophys. J. 2012, 103 (6), 1188–1197. 10.1016/j.bpj.2012.07.054. 22995491 PMC3446717

[ref66] Zhou W.; Stukel J. M.; Cebull H. L.; Willits R. K. Tuning the Mechanical Properties of Poly(Ethylene Glycol) Microgel-Based Scaffolds to Increase 3D Schwann Cell Proliferation. Macromol. Biosci 2016, 16 (4), 535–544. 10.1002/mabi.201500336. 26726886

[ref67] Liu W.; Zhou X.; Mao Z.; Yu D.; Wang B.; Gao C. Uptake of Hydrogel Particles with Different Stiffness and Its Influence on HepG2 Cell Functions. Soft Matter 2012, 8 (35), 9235–9245. 10.1039/c2sm26001h.

