# Supplementary material for: Indenting at the Microscale: Guidelines for Robust Mechanical Characterization of Alginate Microgels
Source: ACS Appl Mater Interfaces. 2025 Feb 24;17(9):13513–26. doi: 10.1021/acsami.4c20952 (PMC11891838; doi:10.1021/acsami.4c20952)
Supplement: Supplementary file 4 — am4c20952_si_004.pdf [file am4c20952_si_004.pdf]

Supporting Information

Indenting at the Microscale: Guidelines for Robust Mechanical Characterization of Alginate Microgels

Philipp Harder[1,2,3], Leonard Funke[1,3], Jana Tamara Reh[3,4,5], Oliver Lieleg[3,4,5], Berna Özkale[1,2,3,]*

[1]Microrobotic Bioengineering Lab (MRBL), School of Computation Information and Technology, Technical University of Munich, 85748, Hans-Piloty-Straße 1, Garching.

[2]Munich Institute of Robotics and Machine Intelligence, Technical University of Munich, Georg-Brauchle-Ring 60, 80992 Munich, Germany.

[3]Munich Institute of Biomedical Engineering, Technical University of Munich, Boltzmannstraße 11, 85748 Garching, Germany.

[4]TUM School of Engineering and Design, Department of Materials Engineering, Technical University of Munich, 85748, Boltzmannstraße 15, Garching, Germany.

[5]Center for Protein Assemblies (CPA), Technical University of Munich, 85748, Ernst-Otto-Fischer-Str. 8, Garching, Germany.

*Corresponding author. Email: berna.oezkale@tum.de

Corresponding Author

*Berna.oezkale@tum.de

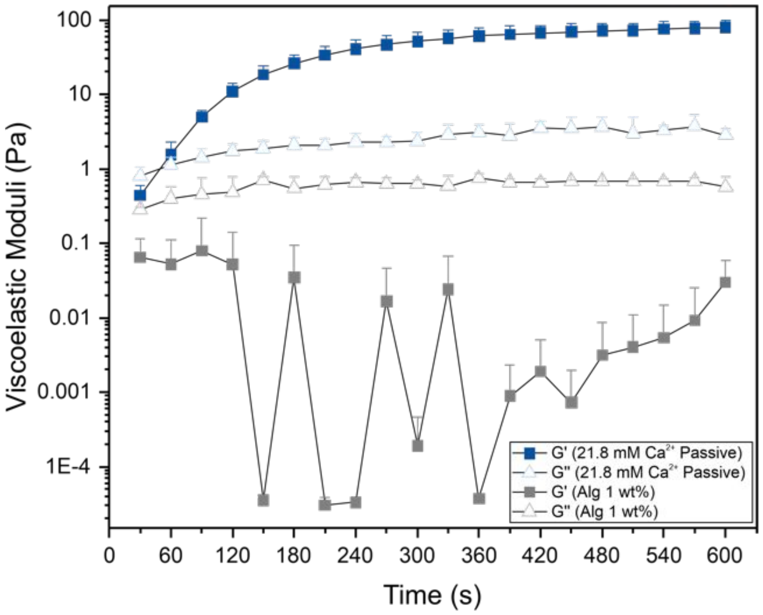

Figure S1: Viscoelastic moduli (1 Hz) of M1-type RhB alginate macrogel pre-polymer mixture. M1-type RhB alginate macrogel, diluted to a final 1 wt%, with and without the addition of $CaCO_3$ nanoparticles (21.8 mM Ca^{2+}) are shown. Moduli were recorded over a 10-minute period (n = 3). For the sample containing $CaCO_3$, *G*’ dominates *G*” After 60 seconds, indicating that a gel is formed only when $CaCO_3$ is added.

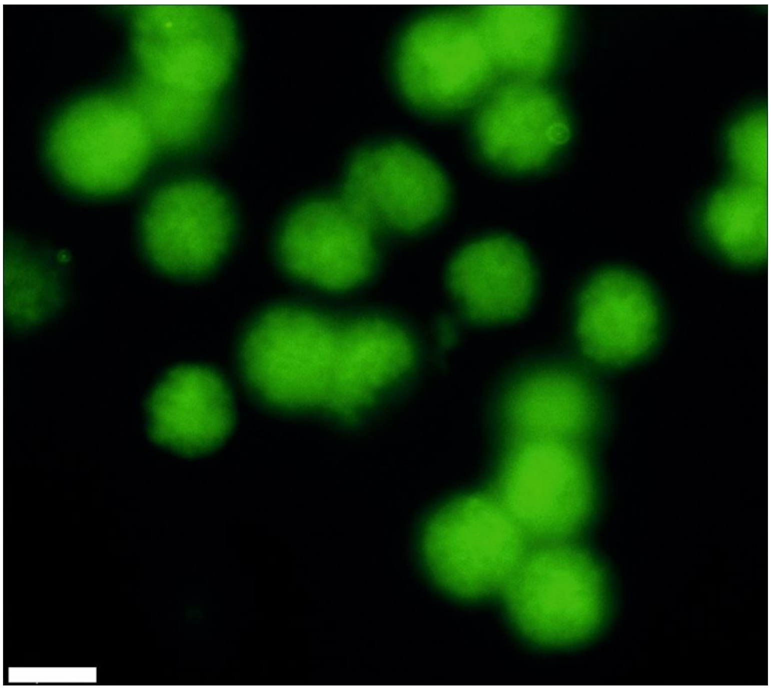

Figure S2: Microgels fabricated with 3.1 mM Ca^{2+} ions. Insufficient crosslinking density leads to poorly formed microgels. Fluorescent images of M1-type RhB-microgels fabricated with 30 µm channel size and 3.1 mM Ca^{2+} crosslinker concentration. Scale bar: 30 µm.

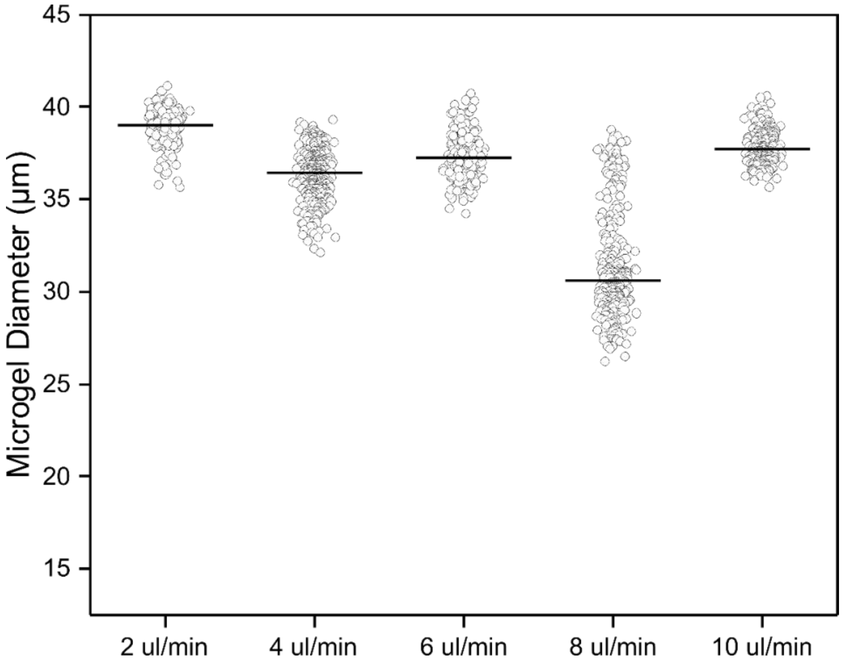

Figure S3: Microgels fabricated with varying flow speeds resulting in size differences. M1-type microgels fabricated in the 30 µm channel, with 21.8 mM $CaCO_3$, at varying flow speeds resulting in a change in size at 8 µl/m.

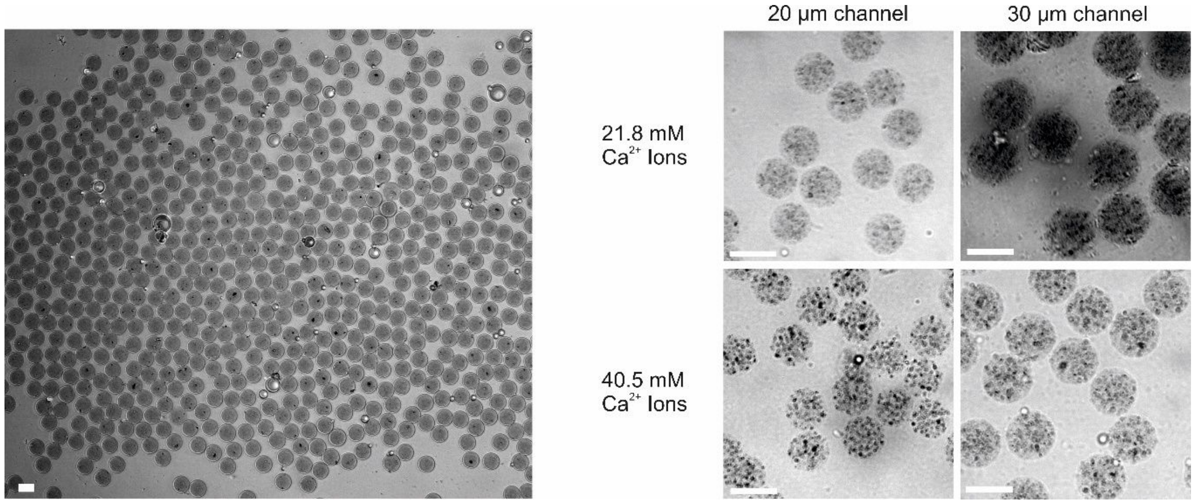

Figure S4: Exemplary images of M2-type, gold nanoparticle carrying, alginate microgels made with 21.8 mM to 40.5 mM Ca^{2+} crosslinker concentrations. Fabricated via 20 µm and 30 µm microfluidic channels. Scale bars: 30 µm.

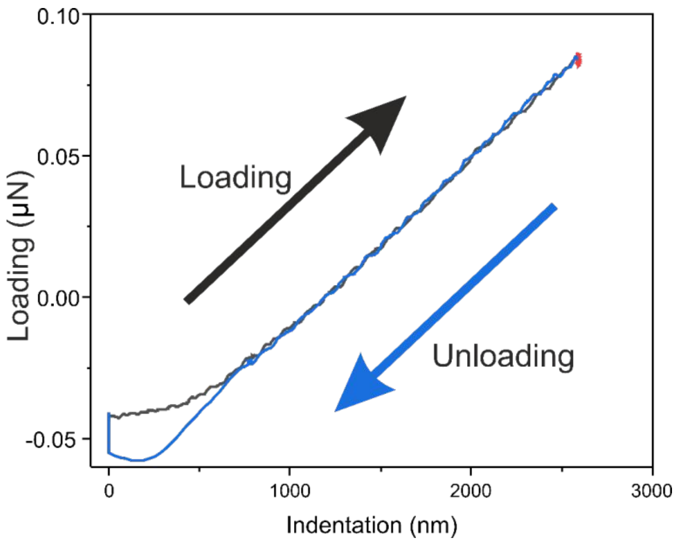

Figure S5: Exemplary nanoindentation load curve. The microgel sample behaves linear elastic at an indentation depth of up to 2600 nm. The black line indicates load direction, while the blue line indicates unloading. A small adhesion process was observed during the end of unloading.

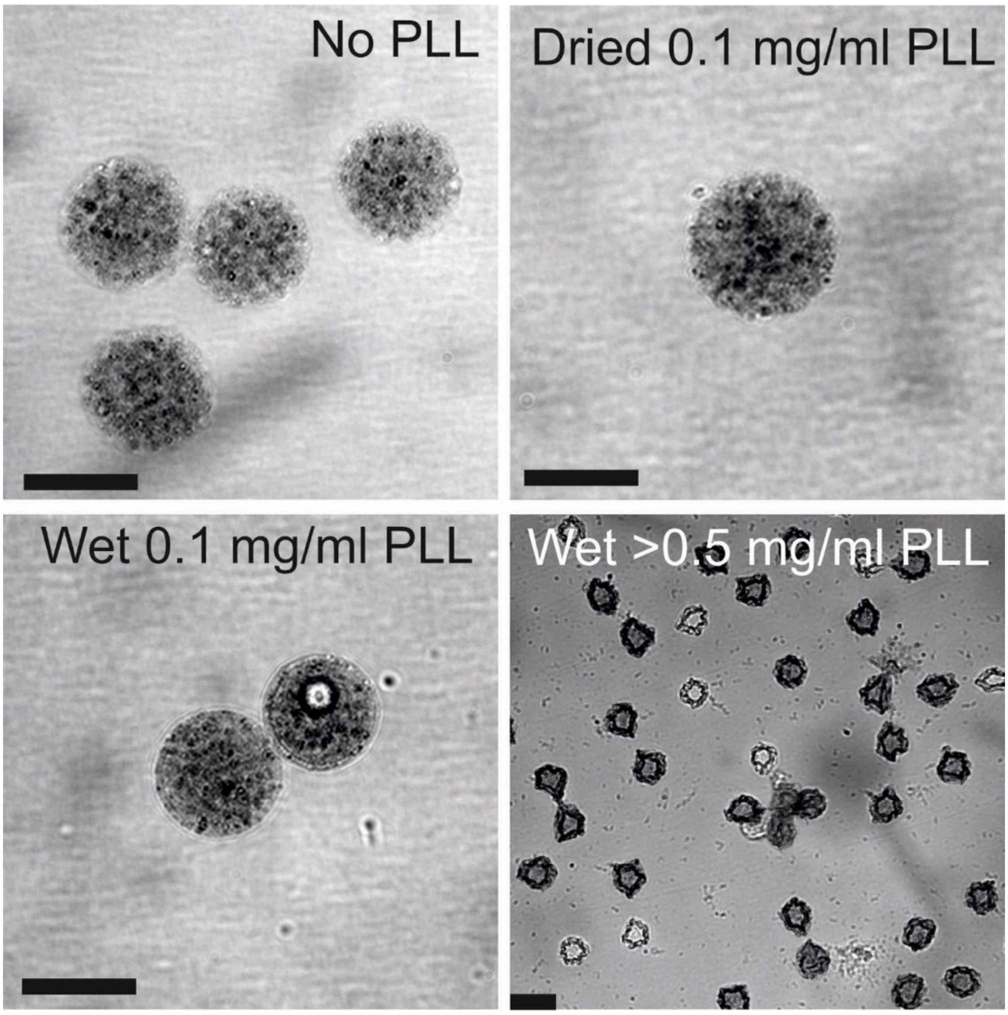

Figure S6: Secondary crosslinking of microgels through high amounts of PLL. Representative images of deformed microgels without PLL, microgels on a substrate treated with 0.1 mg/ml PLL and dried before microgel addition, microgels on a substrate treated with 0.1 mg/ml PLL without drying, and microgels subjected to high concentrations of PLL over 0.5 mg/ml. Scale bars: 30 µm.

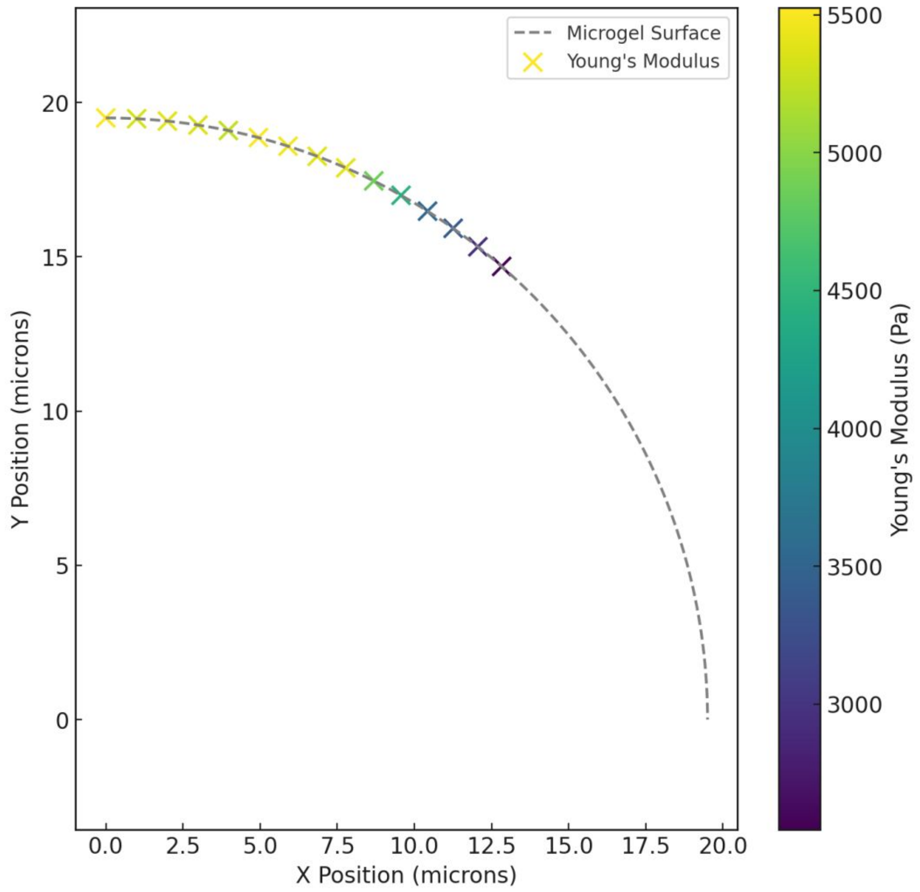

Figure S7: Nanoindenation scan of a single microgel. A single microgel was scanned using nanoindentation with a step size of 1 µm, showing a mean stiffness of 5.4 ± 0.08 kPa for the first eight indentations.

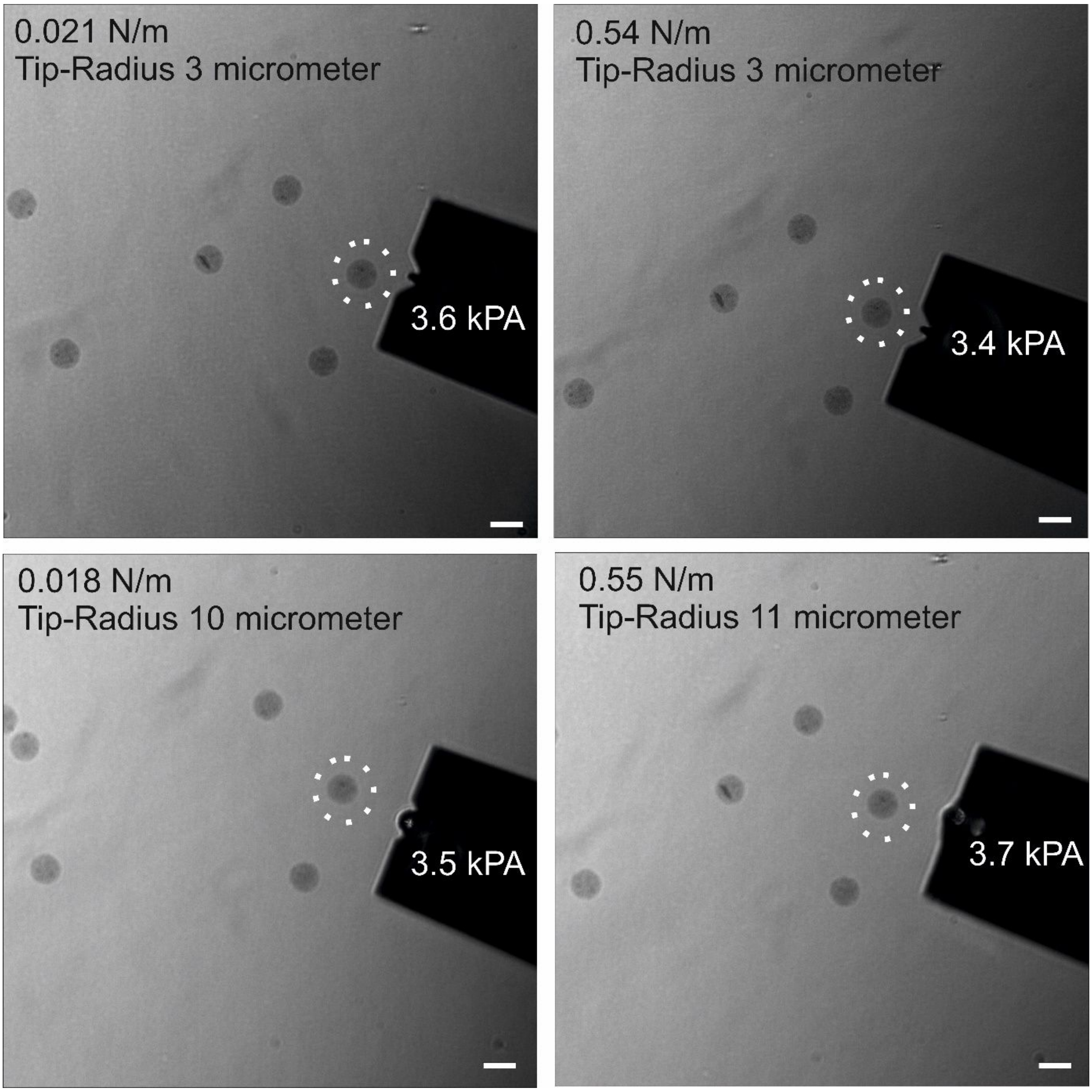

Figure S8: Comparison of different nanoindentation probes used to measure the same microgel. Probes with tip sizes of 3 µm (spring constant: 0.54 N/m), 11 µm (spring constant: 0.55 N/m), 3 µm (spring constant: 0.021 N/m), and 11 µm (spring constant: 0.018 N/m) demonstrated similar measurement results with minor deviations (maximum 0.3 kPa). Scale bars: 30 µm.

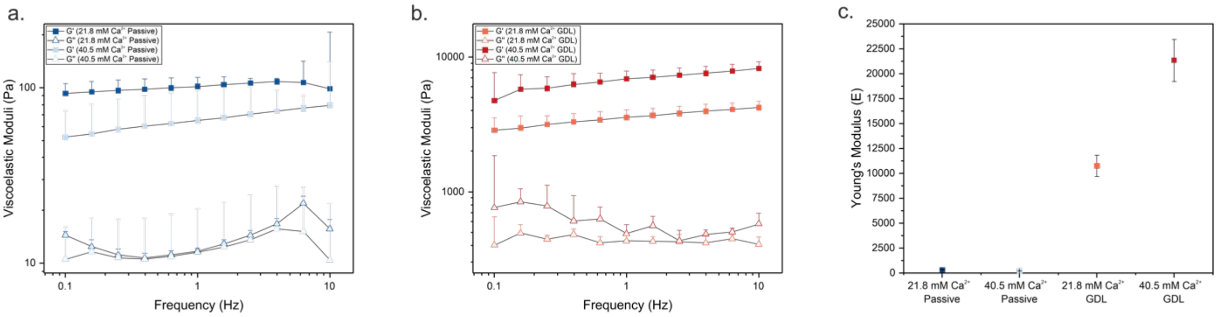

Figure S9: Viscoelastic properties of 21.8 and 40.5 mM Ca^{2+} M1 alginate macrogels and the calculated Young's moduli (E). a) Frequency-dependent viscoelastic moduli of passively crosslinked M1 alginate macrogels without GDL in the prepolymer mixture (21.8 and 40.5 mM Ca^{2+}), according to the formulation used in microfluidic fabrication. Crosslinking was allowed to occur over 12 hours, relying on the passive dissolution of $CaCO_3$ nanoparticles via 0.037 vol% acetic acid in the surrounding media (n = 3). b) Frequency-dependent viscoelastic moduli of M1-type alginate macrogels mixed with $CaCO_3$ nanoparticles (21.8 and 40.5 mM Ca^{2+}) using glucono-δ-lactone (GDL) as a crosslinking agent. The macrogels were allowed to crosslink over 12 hours (n = 3). c) Calculated Young's moduli (E) for macrogels crosslinked via passive diffusion of acetic acid and active initiation of crosslinking via GDL in pre-polymer mixture. Error bars denote the standard deviation.

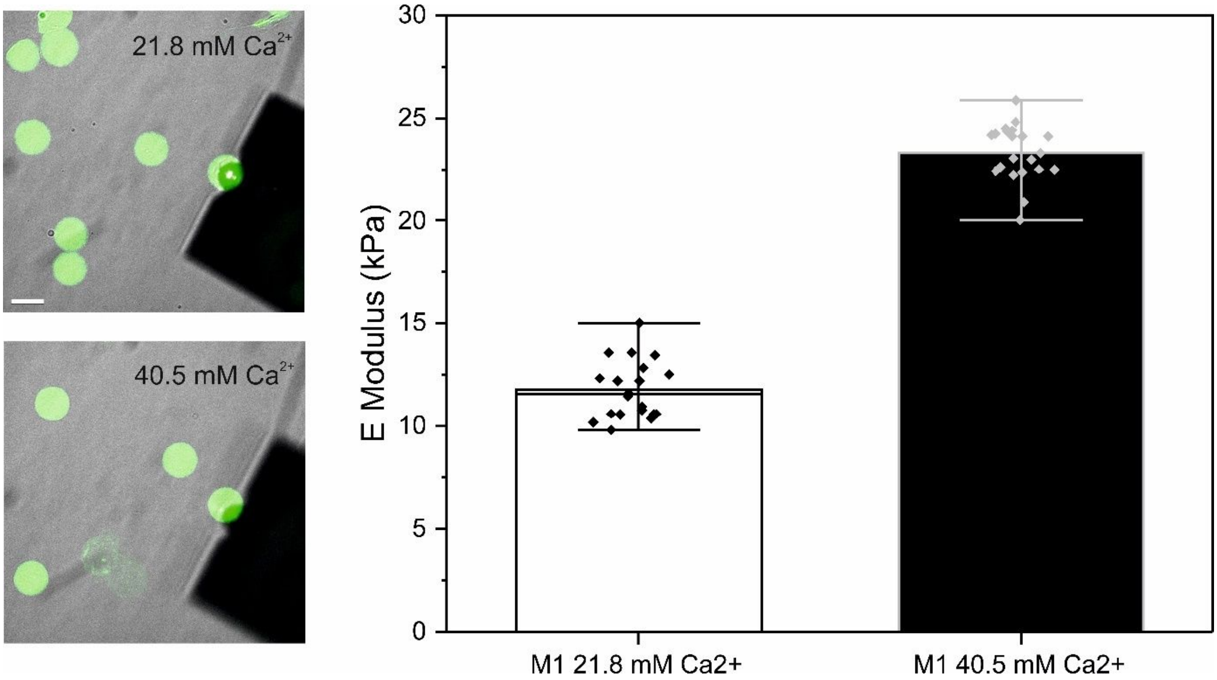

Figure S10: Microscope images and nanoindentation measurements of M1-type microgels. Superimposed brighfield and Rhodamine B fluorescence images of M1 alginate microgels during nanoindentation, with corresponding Young's Moduli (E Moduli) fabricated at 21.8 and 40.5 mM Ca^{2+} crosslinker concentration (n = 21 per condition). Scale bar: 30 µm.

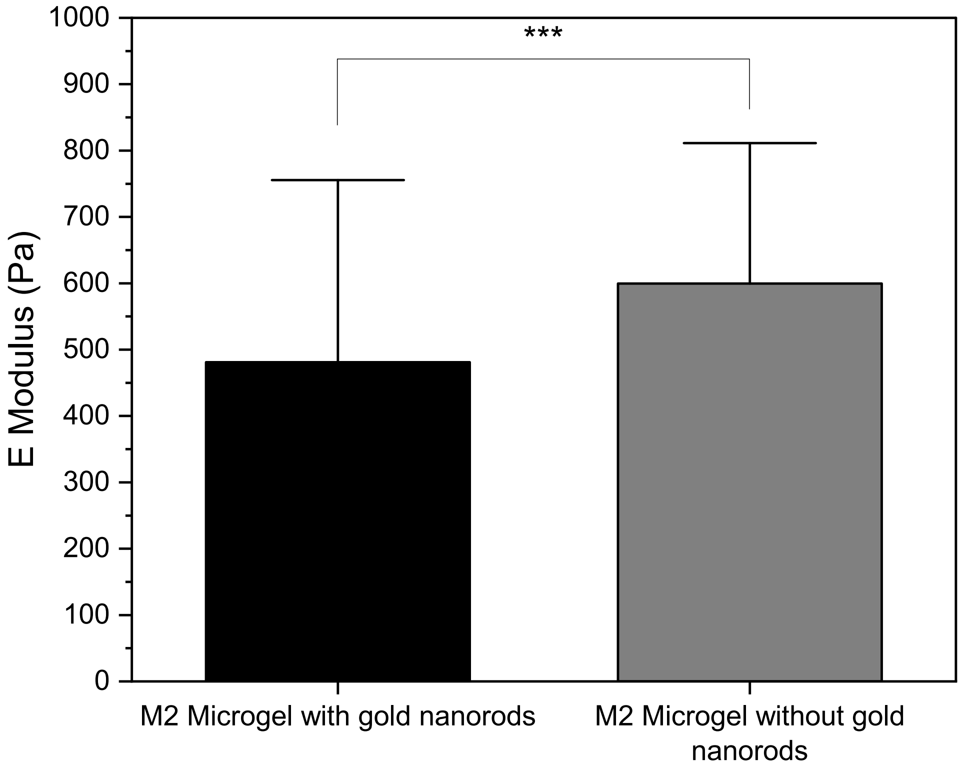

Figure S11: Nanoindentation results of M2-type microgels with and without gold nanorods. The Young's moduli (*E*) of two types of M2-type microgels measured in storage buffer (n>20, *** $p < .001$, Wilcoxon Signed-Rank Test).

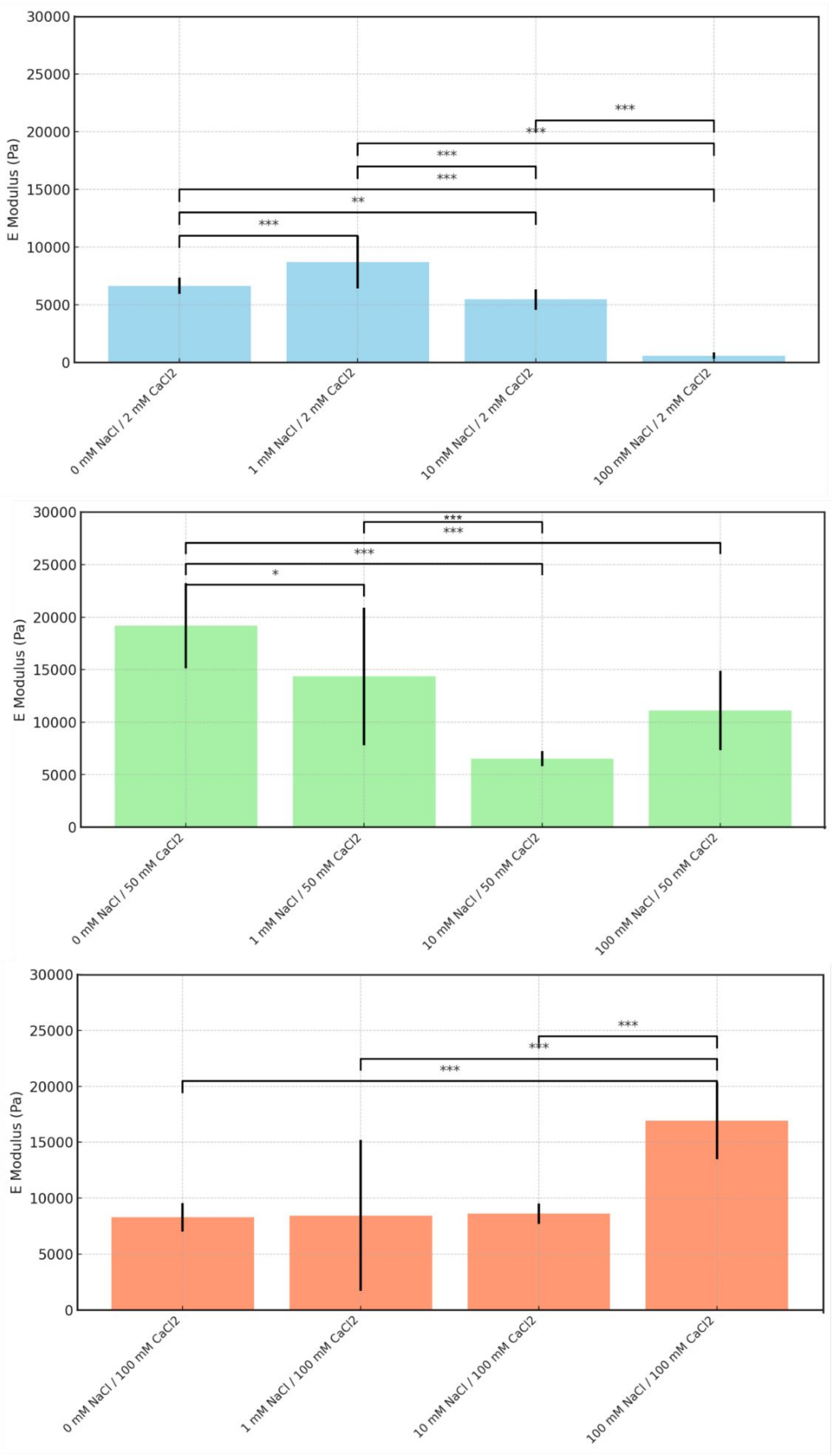

Figure S12: Comparison of Young's moduli (E) of M2-type microgels under different NaCl and $CaCl_2$ conditions. The data are grouped by $CaCl_2$ concentration 2 mM (blue), 50 mM (green), and 100 mM (orange). Each bar represents the mean ± standard deviation based on the number of measurements for each condition. Statistical analysis was conducted using one-way ANOVA within each group to evaluate

differences in stiffness across NaCl concentrations. Pairwise comparisons were performed using Tukey's HSD test to identify significant differences between conditions. Significant differences are indicated by bars and corresponding significance levels (* $p < .05$,** $p < .01$, *** $p < .001$, ANOVA).

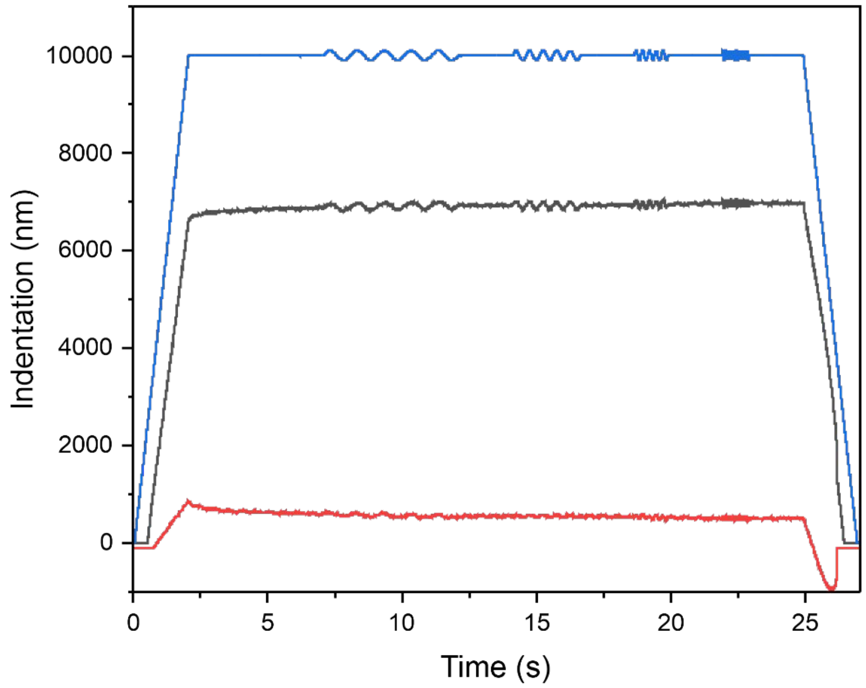

Figure S13: Exemplary nanoindentation load curve using DMA. Blue indicates the Piezo movement, black is the cantilever movement, and red is the indentation into the M2 microgel. Dynamic loading was conducted at 1 Hz, 2 Hz, 4 Hz, and 10 Hz.

Video S1: Undesirable microgel motion during indentation without PLL coating on the substrate.

Video S2: Stable nanoindentation procedure in mapping mode over the microgel surface, enabled by secure attachment of microgel to the substrate surface.

Video S3: Change in microgel integrity under increasing concentrations (1, 10, and 20 mM) of NaCl.
